# Acidosis induces reprogramming of cellular metabolism to mitigate oxidative stress

**DOI:** 10.1186/2049-3002-1-23

**Published:** 2013-12-23

**Authors:** Gregory LaMonte, Xiaohu Tang, Julia Ling-Yu Chen, Jianli Wu, Chien-Kuang Cornelia Ding, Melissa M Keenan, Carolyn Sangokoya, Hsiu-Ni Kung, Olga Ilkayeva, László G Boros, Christopher B Newgard, Jen-Tsan Chi

**Affiliations:** 1 Institute for Genome Sciences & Policy, Durham, NC, USA; 2 Department of Molecular Genetics & Microbiology, Durham, NC, USA; 3 Department of Anatomy and Cell Biology, School of Medicine, National Taiwan University, Taipei, Taiwan; 4 Sarah W Stedman Nutrition and Metabolism Center, Durham, NC, USA; 5 Duke Institute of Physiology, Durham, NC, USA; 6 Department of Pediatrics, University of California Los Angeles School of Medicine, Los Angeles, CA, USA; 7 LABIOMED & SiDMAP, LLC, Torrance, CA, USA; 8 Department of Pharmacology and Cancer Biology, Duke University, Durham, NC, USA

## Abstract

**Background:**

A variety of oncogenic and environmental factors alter tumor metabolism to serve the distinct cellular biosynthetic and bioenergetic needs present during oncogenesis. Extracellular acidosis is a common microenvironmental stress in solid tumors, but little is known about its metabolic influence, particularly when present in the absence of hypoxia. In order to characterize the extent of tumor cell metabolic adaptations to acidosis, we employed stable isotope tracers to examine how acidosis impacts glucose, glutamine, and palmitate metabolism in breast cancer cells exposed to extracellular acidosis.

**Results:**

Acidosis increased both glutaminolysis and fatty acid β-oxidation, which contribute metabolic intermediates to drive the tricarboxylic acid cycle (TCA cycle) and ATP generation. Acidosis also led to a decoupling of glutaminolysis and novel glutathione (GSH) synthesis by repressing *GCLC/GCLM* expression. We further found that acidosis redirects glucose away from lactate production and towards the oxidative branch of the pentose phosphate pathway (PPP). These changes all serve to increase nicotinamide adenine dinucleotide phosphate (NADPH) production and counter the increase in reactive oxygen species (ROS) present under acidosis. The reduced novel GSH synthesis under acidosis may explain the increased demand for NADPH to recycle existing pools of GSH. Interestingly, acidosis also disconnected novel ribose synthesis from the oxidative PPP, seemingly to reroute PPP metabolites to the TCA cycle. Finally, we found that acidosis activates p53, which contributes to both the enhanced PPP and increased glutaminolysis, at least in part, through the induction of *G6PD* and *GLS2* genes.

**Conclusions:**

Acidosis alters the cellular metabolism of several major metabolites, which induces a significant degree of metabolic inflexibility. Cells exposed to acidosis largely rely upon mitochondrial metabolism for energy generation to the extent that metabolic intermediates are redirected away from several other critical metabolic processes, including ribose and glutathione synthesis. These alterations lead to both a decrease in cellular proliferation and increased sensitivity to ROS. Collectively, these data reveal a role for p53 in cellular metabolic reprogramming under acidosis, in order to permit increased bioenergetic capacity and ROS neutralization. Understanding the metabolic adaptations that cancer cells make under acidosis may present opportunities to generate anti-tumor therapeutic agents that are more tumor-specific.

## Background

Tumor metabolism is essential to meet the various metabolic demands associated with the proliferation and expansion of tumor cells. These needs fall into several categories, including: (i) bioenergetics (ATP and other energy equivalents required for energy homeostasis); (ii) biosynthetic (biochemical intermediates required for the synthesis of macromolecules for cell proliferation such as fatty acids and nucleotides); and (iii) reductive capacity (for example, nicotinamide adenine dinucleotide phosphate (NADPH)) for a variety of biochemical reactions to neutralize increased reactive oxidative stress (ROS). Perhaps the most prominent example of altered tumor metabolism is the increased glucose utilization and excessive production of lactic acid in many solid tumors, termed the ‘Warburg’ effect [1]. The continuous expansion of tumor cells beyond the capacity of local vascular perfusion leads to a variety of environmental alterations, such as oxygen depletion (hypoxia), glucose deprivation, high lactate levels (lactosis), and extracellular acidosis [2-8]. These microenvironmental stresses exert important influences on gene expression and metabolic phenotypes. For example, hypoxia induces expression of genes involved in the transport and metabolism of glucose [9,10] while restricting the entry of metabolites into the mitochondria by activating pyruvate dehydrogenase kinase (*PDK*) [11,12]. Hypoxia also renders cells reliant on the reductive carboxylation of glutamine-derived α-ketoglutarate (α-KG) for *de novo* lipogenesis [13,14]. Compared with hypoxia, relatively little is known about the metabolic adaptations in response to acidosis. Previous studies have suggested that acidosis can influence hypoxia responses, inhibit glycolysis and trigger autophagy [5,6,15,16]. In renal cells, metabolic acidosis increases glutamine and glutamate metabolism [17,18]. However, many details about the cellular metabolic reprogramming under acidosis remain unknown.

NADPH plays a crucial role in the defense against ROS and reductive biosynthetic reactions (for example, lipogenesis) to fuel macromolecular biosynthesis. One key source of NADPH is the pentose phosphate pathway (PPP), by which glycolysis intermediates (for example, glucose-6-phosphate (G6P)) are used to generate NADPH and ribose-5-phophsate (R5P). The PPP possesses an oxidative and non-oxidative branch, both of which have been demonstrated to be activated or overexpressed in human cancer. In the oxidative branch, glucose-6-phosphate dehydrogenase (*G6PD* or *G6PDH*) is the first and rate-limiting enzyme, non-reversibly oxidizing G6P to 6-P-gluconolactone, a reaction which generates NADPH. G6PD deficiency in humans impairs the generation of NADPH, leading to significant red cell lysis and anemia due to oxidative stress. The non-oxidative branch of the PPP is mediated by the reversible reactions of several transketolases (*TKT, TKTL1, TKTL2*) and transaldolase, which generate the sugar moiety of the nucleotide precursor R5P for nucleotide synthesis during proliferation. Since the non-oxidative PPP is reversible, any excess amount of pentose phosphate can be converted back to glycolysis when bioenergetic (ATP) requirements exceed biosynthetic need (for example, R5P). Beside glucose, both glutamine and several fatty acids (via acetyl-CoA) can function as substrates for various bioenergetic and biosynthetic processes. Glutamine in particular serves as a substrate for a variety of cellular processes, including citrate synthesis and lipogenesis [13,14], *de novo* synthesis of glutathione, and as carbon source for the canonical tricarboxylic acid cycle (TCA cycle) via α-KG.

Recently, various mass-spectrometry-based metabolomics techniques have been used to measure the steady-state levels of metabolites and quantitate metabolic flux [19,20]. These approaches have identified metabolites associated with tumor progression [19-21] and the process of reductive carboxylation of glutamine to citrate [14]. No similar approach has yet been applied to define the effect of acidosis. Here, we applied stable-isotope tracer measurements to define how acidosis affects cellular metabolism. Together with measurements of intracellular amino acids and transcriptional profiling of cancer cells under acidosis [6], these data reveal extensive metabolic reprogramming and critical metabolic adaptations that offer the opportunity to target cancer cells selectively under acidosis.

## Methods

### Cell culture and modeling various microenvironmental stresses

MCF-7, ZR-75-1, T47D, MDA-MB-231 and MDA-MB-157 cells were cultured in RPMI with 2.0 g/l glucose, 10% fetal bovine serum, 1 × antibiotics (penicillin, 10,000 UI/ml; streptomycin, 10,000 UI/ml) and 25 mM 2-[4-(2-hydroxyethyl)piperazin-1-yl]ethanesulfonic acid (HEPES). Cell lines, obtained from and initially validated by the Duke Cell Culture Facility (Durham, NC, USA), were maintained for fewer than 6 months and validated by microscopy every 1 to 2 days. Lactosis was generated via addition of sodium lactate (Sigma), while acidosis was generated via media pH adjustment to pH 6.7 by HCl. For the α-KG rescue experiments, media was supplemented with 700 μM dimethyl α-KG (Sigma-Aldrich (St. Louis, MO, USA). The paired shp53 and shControl MCF-7 cells were as previously described [22]. Control or gene-specific small interfering (si)RNAs (Additional file 1: Table S1; Ambion/Life Technologies (Grand Island, NY, USA)) were transfected using lipofectamine 2000 in OPTIMEM according to the manufacturer’s instructions. The cDNA expression constructs for nuclear factor erythroid 2-related factor 2 (NRF2) were purchased from Origene (Rockville, Md, USA) and described previously [23], while cDNA expression constructs for GLS2 were a generous gift of Dr Zhaohui Feng from the Robert Wood Johnson Medical School (New Brunswick, NJ, USA) [24]. The efficiency of silencing was determined by western blots.

### Stable isotope labeling experiments

A total of 2 × 10^6^ MCF-7 cells were plated in 10 cm dishes in quadruplicate. After 24 h, the media was changed to media containing either 2 g/l glucose (50% (1 g/l) (1,2-^13^C_2_)d-glucose and 50% (1 g/l) unlabeled glucose), 2 mM (100% (uniformly-labeled U-^13^C)) glutamine or 10 μM (U-^13^C) palmitate. Cells were exposed to control or acidosis (pH 6.7) media for 24 h. Culture medium (for CO_2_, glucose, glutamate, and lactate isotopomer measurements) and cell pellets (for palmitate and ribose measurements) were collected after cells were washed twice in 1 × PBS, harvested via cell scraping on ice, and specific extractions were performed as described below and as previously reported [25].

The procedures for extraction and derivatization of glucose, cholesterol, ribose, fatty acids, lactate, CO_2_ and glutamate have been previously published [26,27]. Fatty acids were extracted by saponification of Trizol (500 μl, Invitrogen, Carlsbad, CA, USA) cell extract, after removal of the upper glycogen-containing and RNA-containing supernatant, using 30% KOH and 70% ethanol (300 μl each) for 2 h. Fatty acids were extracted by further acidification using 6 N hydrochloric acid to a pH below 2.0 and repeated vortexing with 5 ml petroleum ether. Fatty acids (palmitate) were monitored at m/z 270, using canola oil as positive control. The enrichment of acetyl units in media and cell pellet palmitate in response to acidosis was determined using the mass isotopomer distribution analysis (MIDA) approach. Acetyl-CoA and fractions of new synthesis were calculated from the M4/M2 ratio using the formula M4/M2 = (n - 1)/2•(p/q), where n is the number of acetyl units, p is the ^13^C labeled precursor acetate fraction and q is the ^12^C-labeled natural acetate fraction (p + q = 1) [28].

For glucose extraction, 500 μl each of 0.3 N barium hydroxide and 0.3 N zinc sulfate were added to 100 μl media. Samples were vortexed and centrifuged for 15 minutes at 10,000 rpm. The supernatant was then dried on air over heat and were derivatized by adding 150 μl hydroxylamine solution and incubated for 2 h at 100°C followed by addition of 100 μl of acetic anhydride. Samples were incubated at 100°C for 1 h and dried under nitrogen over heat as previously described in the fatty acids derivatization section. Ethyl acetate (200 μl) was added. Peak glucose ion was detected at the m/z 187 cluster.

Lactate was extracted from media through acidification of 100 μl media with HCl and addition of 1 ml of ethyl acetate. The resulting aqueous layer was dried under nitrogen over heat and derivatized using lactate standard solution as positive control. A total of 200 μl of 2,2-dimethoxypropane was added followed by 50 μl of 0.5 N methanolic HCl. Samples were incubated at 75°C for 1 h. Then, 60 μl of *n*-propylamine was added and samples were heated for 100°C for 1 h followed by addition of 200 μl dichloromethane. Heptafluorobutyric anhydride (15 μl) was added followed by 150 μl of dichloromethane and samples were subjected to gas chromatography/mass spectrometry (GC/MS). M1 and M2 lactate were differentiated to distinguish the pentose phosphate flux from anaerobic glycolysis [25,28] and the ion cluster at m/z 328 was examined.

Media glutamate was converted into its *n*-trifluoroacteyl-*n*-butyl derivative and monitored at ion clusters at m/z152 and m/z198. ^13^CO_2_ Assay for CO_2_ was generated by adding equal volumes (50 μl) of 0.1 N NaHCO_3_ and 1 N HCl to spent media and ^12^CO_2_/^13^CO_2_ ion currents were monitored and calculated from the m/z44 and m/z45 peak intensities, respectively, using ^13^CO_2_/^12^CO_2_ of in house cell culture cabinet’s CO_2_ tank as the reference ratio for ^13^CO_2_ Δ calculations. This ratio of ^13^CO_2_/^12^CO_2_ was determined with gas chromatography-mass spectrometry (Agilent, Palo Alto, CA, USA, 5975 MS and 6890 N (network) GC system for volatile (gas) isotopomer data acquisitions,) as previously described [29].

Isolation of RNA ribose was performed as previously reported [30]. Briefly, RNA ribose was first isolated by acid hydrolysis of cellular RNA after Trizol purification from cell pellets. Total RNA abundance was then quantified by spectrophotometric determination in quadruplicate. Cellular ribose was derivatized to its aldonitrile acetate form using hydroxylamine, resuspended in pyridine with acetic anhydride (Supelco/Sigma-Aldrich, St. Louis, MO, USA) before mass spectral analyses. The ion cluster was measured around *m/z* 256 (carbons 1 to 5 of ribose; chemical ionization (total ribose)), *m/z* 217 (carbons 3 to 5 of ribose (non-oxidative branch ribose)), and *m/z* 242 (carbons 1 to 4 of ribose; electron impact ionization (oxidative branch ribose)) to determine molar enrichment and the positional distribution of ^13^C in ribose.

An Agilent (Palo Alto, CA, USA) 5975 Inert XL Mass Selective Detector connected to HP6890N network gas chromatograph was used to detect mass spectral data under the following settings: GC inlet 230°C, MS source 230°C, MS quad 150°C [27]. For media CO_2_, glucose, lactate and glutamate analyses, an HP-5 column (30 m length × 250 μm diameter × 0.25 μm thickness) was used while a DB-23 column (60 m length, 250 μm diameter × 0.15 μm thickness) was used for fatty acid measurement.

Statistics for mass spectral analyses were obtained by consecutive and independent injections of 1 μl sample using an autosampler with optimal split ratios for column loading (10^6^ > abundance > 10^4^ abundance). Data was accepted if the standard sample deviation was below 10% of the normalized peak intensity (integrated peak area of ion currents; 100%) among repeated injections. Data download was performed in triplicate manual peak integrations using modified (background subtracted) spectra under the overlapping isotopomer peaks of the total ion chromatogram (TIC) window displayed by the Chemstation (Agilent, Palo Alto, CA, USA) software. A two-tailed independent sample t test was used to test for significance (*P <0.05, **P <0.01) between control and treated groups.

Rapid system-wide association study (SWAS) evaluation of MCF-7 cells was performed by the color assisted visual isotopolome data matrix screening tool [26], to diagnose phenotypic differences and response to acidosis. The isotope labeled fractions, after subtracting natural ^13^C enrichment, of all metabolic products from the ^13^C tracer were found in total ion currents, obtained by chromatography separation in the selected ion monitoring (SIM) mode. These SIMs included all isotope labeled products with the range that covers all possible single and multiple substitutions, based on the number of carbons making up the reported biomolecules and their fragment. The sum of all labeled isoforms were then generated by the number of ^13^C substitutions, expressed as labeled fraction, of which positional ^13^C isoforms were normalized to 100% and expressed as fractions (Σμ) of the ^13^C labeled portion of the molecule. The sum of all labeled isoforms was also weighed by the number of ^13^C position, expressed as ^13^C content (Σμ_ν_). This number is also known as total activity (isotope accumulation) when radiating isotopes are used.

### Amino-acid profiling

The measurement of intracellular amino acids was performed using stable isotope dilution techniques, flow injection tandem mass spectrometry and sample preparation methods described previously [31,32]. Quantification was facilitated by the use of stable isotope internal standards as published [32].

### RNA isolation and real-time PCR analysis

RNA was extracted using the miRVANA kit (Ambion). A total of 1 μg of total RNA was reverse transcribed by SuperScript II (Invitrogen) for real-time PCR with Power SYBRGreen Mix (Applied Biosystems/Life Technologies (Grand Island, NY, USA)) and primers for indicated genes (Additional file 1: Table S1).

### Cell viability assays

Cell viability was primarily evaluated by direct cell counting (trypan blue exclusion), propidium iodide (PI) and 3-[4,5-dimethylthiazol-2-yl]-2,5 diphenyl tetrazolium bromide (MTT) assays (Promega (Madison, WI, USA)). For direct cell counting, 48 h after siRNA transfection, stresses were applied (T = 0) and cells were counted at the indicated timepoints, with two separate counts averaged after trypan blue exclusion. For PI, cells were washed twice with 1 × PBS, frozen at -80°C, thawed then treated with 1:10 PI (5 μg/μl) for 30 minutes then absorbance measured at 570 nm. For MTT assays, MTT (5 μg/ml) was added to cell media at 1:10 for 3 h. Afterwards, media was removed and cells resuspended in dimethylsulfoxide (DMSO), then absorbance was measured at 570 nm. The impact of lactic acidosis and acidosis on viability are normalized against corresponding control samples for each time point.

### NADP+/NADPH, glutathione (GSH)/glutathione disulfide (GSSG), and total GSH measurements

NADP^+^/NADPH measurements were performed using the NADP^+^/NADPH and/or GSSG/GSH ratiometric kit (AAT Bioquest (Sunnyvale, CA, USA)) according to suggested protocols. Total GSH was measured using the GSH-Glo kit (Promega (Madison, WI, USA)) according to suggested protocols. Cells were plated and siRNA knockdowns were performed as stated. After 24 h of exposure to neutral (pH 7.4) or acidic (pH 6.7) conditions, cells were lysed and stored at -80°C. Cell extracts were treated with respective NADP+/NADPH, GSH/GSSG, or GSH extraction buffers. The fluorescence was measured at 530/590 nm (for NADPH) or 490/530 (for GSSG), while total GSH was measured by luminescence, and standardized using known GSH concentrations.

### Glutamine uptake

MCF7 cells were plated in 6-well/12-well plates at the density of 800,000/200,000 cells per well. Once cells reached more than 75% confluence, they were washed with 1× PBS twice and then treated under the respective conditions for the indicated time. Cells were then washed with 37°C Krebs-Ringer-HEPES (KRH) buffer twice, followed by the addition of 500 μl/200 μl KRH buffer containing 0.5 μCi/0.2 μCi ^14^C-glutamine (Perkin Elmer (Waltham, MA, USA)) for 1 h at 37°C, and washed three times with 1 ml/400 μl of ice-cold KRH buffer containing 20 mM glutamine to quench the glutamine uptake. Finally, cells were lysed with 1 ml/400 μl radioimmunoprecipitationassay (RIPA) buffer and the lysates were subjected to liquid scintillation counting, and normalized by protein concentrations, measured with Bradford assay.

### G6PD enzyme activity

G6PD enzyme activity based on resazurin was adapted from [33]. Cells were plated for 24 h then lysed using triton-X in tandem with G6PD reaction buffer. G6PD activity was measured via fluorescence at 470/530 nm, blanked against PBS only and normalized against control conditions.

### ROS measurements

Cellular ROS were measured by normalized luminescence using the Oxiselect ROS detection assay (Cell Biolabs (San Diego, CA, USA)). Cells were plated overnight, incubated for 30 minutes at 37°C with the 2’,7’-dichlorodihydrofluorescin (DCFH) dye and then lysed to measure luminescence at 490/530 nm against a DCF standard curve.

### Western blot analysis

MCF7 cells were washed twice with cold 1 × PBS after 24 h of stress and lysed by RIPA buffer. Then, 15 μg of lysates were separated in 10% gels and probed by the indicated antibodies for p53, G6PD, TKT (Cell Signaling (Danvers, MA, USA)), GS (Abnova (Taipei, Taiwan)) and GLS2 and β-tubulin (Cell Signaling (Danvers, MA, USA)). Densitometry was generated using ImageJ (http://rsbweb.nih.gov/ij/).

### Measurements of glutamine, glutamate and ATP

The intracellular glutamine and glutamate levels in cell lysates were measured using the glutamine/glutamate detection kit (Sigma) and normalized by protein contents. For the measurements of media glutamine, media was immediately removed before washing cells and stored overnight at -80°C, then measured as above. The culture media used typically lack glutamate. For ATP measurements, cells were lysed with 100 μl assay reagent, incubated for 5 minutes in the dark and ATP measured using the one-step ATP-lite kit (Perkin-Elmer (Waltham, MA, USA)), then normalized to protein content.

### Statistical analyses

The effects of each stress on gene expression and metabolites (on a log2 scale) were normalized to the control through zero transformation by subtracting the expression levels of the control samples from stressed samples. Error bars for the stable-isotope tracer figures are standard deviations from the mean. For all other graphs, samples sizes are indicated in the figure legends; *P* values were derived by two-tailed t test except for cell growth assays, which were calculated by two-way ANOVA. These were followed, in the event of a significantly different interaction term (between siControl + acidosis versus siRNA + acidosis), by a pairwise comparison using a two-tailed t test. *P* values are indicated (*P ≤0.05, **P ≤0.001, and ***P ≤0.0001) and error bars are standard errors of the mean.

## Results

### The influence of acidosis on glucose metabolism

While previous studies have indicated that both acidosis and lactic acidosis lead to discrete cellular metabolic alterations that are distinct from hypoxia [6], significant details about these processes remain unknown. To thoroughly define the metabolic response to extracellular acidosis, we performed stable-isotope tracing experiments on MCF-7 breast cancer cells exposed to control (pH 7.4) or acidic (pH 6.7) conditions for 24 h. We focused on acidosis, rather than lactic acidosis, to avoid any potential complications resulting from the cellular use of lactate as a metabolic substrate. In addition, we chose a pH of 6.7 since that is within the range (approximately 6.5 to 7.0) of acidity that has been observed in human tumors [34,35]. We cultured MCF-7 cells in quadruplicate with each of three isotope tracers: glucose (1,2^13^C-glucose), glutamine (uniformly labeled (U)^13^C glutamine) and palmitate (U^13^C palmitate). After 24 h, we harvested both cells and culture media to compare the fate of various isotopomers from each isotope tracer under control and acidosis.

**Figure 1:**
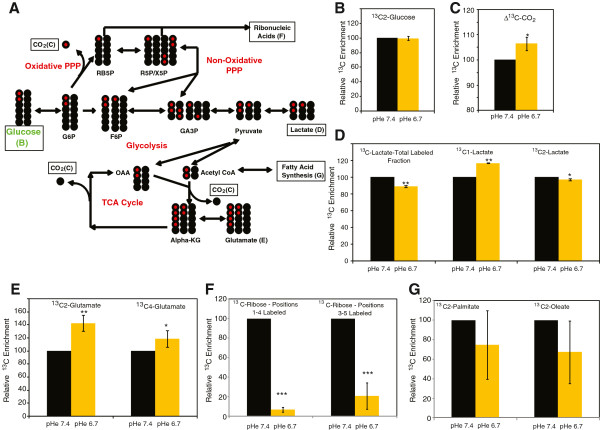
**The use of **^**13**^**C glucose isotope tracers to analyze the glucose metabolism under acidosis. (A)** Schematic graph indicating various measured metabolites (and corresponding panels) in major metabolic pathways resulting from the 1,2^13^C labeled glucose tracer (green). ^13^C labeled and unlabeled carbons are indicated in red and black, respectively. **(B-G)** Relative ^13^C enrichment under control or acidosis conditions for glucose **(B)**, CO_2_**(C)**, lactate **(D)**, glutamate **(E)**, ribonucleic acids **(F)**, and fatty acids **(G)**. Lactate **(D)** is presented as the total ^13^C labeled lactate pool as well as 1 (C1) and 2 (C2) labeled carbon subpools. Glutamate **(E)** is presented as both the 2 (C2) and 4 (C4) labeled subpools. Ribonucleic acids **(F)** are presented as the ^13^C positions 1 to 4 and ^13^C positions 3 to 5 subpools. Fatty acids **(G)** are presented as 2-carbon ^13^C-labeled palmitate and oleate. Error bars are mean ± SD, significant *P* values are indicated (*P ≤0.05, **P ≤0.01, ***P ≤0.001).

From the isotope-labeled 1,2^13^C^2^-glucose, we compared several metabolites (Figure 1A) including CO_2_, lactate, glutamate, ribonucleic acids and fatty acids (palmitate and oleate) under control and acidosis conditions (the relative changes are shown in Figure 1, with the complete data, provided as ONCOisobolome matrix tables, in Additional file 1: Tables S2 and S3). We observed an equal level of labeled medium glucose after 24 h (Figure 1B), suggesting equal availability during the experimental periods. Compared with control, acidosis increased the abundance of total labeled ^13^C-CO_2_ being produced from glucose (Figure 1C). We also noted a relative decrease in the total labeled lactate and ^13^C^2^-labeled lactate, but an increase in ^13^C^1^-labeled lactate (Figure 1D). Since C^1^ is oxidized to CO_2_ in the PPP, the increase in C^1^-lactate, compared to C^2^-labeled lactate suggests that glucose is increasingly redirected to the PPP under acidosis. This is consistent with an increase in the labeled CO_2_, where the PPP is the most likely source (Figure 1C). The observed decrease in total labeled lactate is consistent with previous reports of decreased glycolysis under acidosis [5,6]. Acidosis also increased ^13^C^2^-labeled and ^13^C^4^-labeled glutamate (Figure 1E), which results from one (^13^C^2^-labeled) or two (^13^C^4^-labeled) rounds of the TCA cycle. Increased glutamate labeling from glucose under acidosis indicates increased glucose conversion to pyruvate, which can enter the TCA cycle, consistent with an increased reliance upon mitochondrial metabolism under acidosis [5,6]. Interestingly, we also observed a >90% reduction in ribose synthesis from glucose (Figure 1F), suggesting that acidosis abolished most of the biosynthetic conversion of glucose to ribonucleotides. Finally, we observed an insignificant decrease in labeled fatty acids under acidosis (Figure 1G), suggesting no major changes in fatty acid synthesis.

These results suggest that glucose metabolism is extremely sensitive to acidosis. Our findings confirm several previously reported metabolic adaptations to acidosis, namely increased TCA cycle metabolism (increased labeled glutamine) and decreased glycolysis (decreased total labeled lactate production). More striking, however, is the induction of the oxidative branch of the PPP, coupled with a dramatic decrease in ribose labeling from glucose. While the decrease in total ribonucleic acid labeling may simply indicate decreased cellular proliferation under acidosis, the induction of glucose to the oxidative branch of the pentose phosphate pathway (indicated by C^1^-labeled lactate (Figure 1D)) indicates an increased need for reductive potential under acidosis. Glucose intermediates from the oxidative PPP represent the building blocks of ribose synthesis, so the disconnect between increased oxidative PPP activity (Figure 1D) and reduced ribose synthesis (Figure 1F) suggests that the metabolic intermediates of the non-oxidative branch may be drawn away from ribose synthesis towards glycolysis and the TCA cycle.

### The influence of acidosis on palmitate and glutamine metabolism

**Figure 2:**
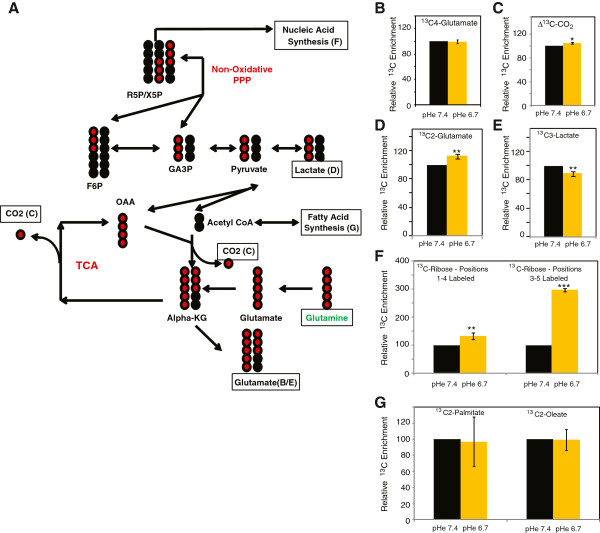
**The use of **^**13**^**C glutamine isotope tracer to analyze glutamine metabolism under acidosis. (A)** Schematic graph indicating various measured metabolites (and corresponding panels) resulting from the uniformly ^13^C labeled glutamine tracer (green). The ^13^C labeled and unlabeled carbons are indicated in red and black, respectively. **(B-G)** Relative ^13^C enrichment in the glutamate **(B,D)**, CO_2_**(C)**, lactate **(E)**, ribonucleic acids **(F)** and fatty acids **(G)** under control or acidosis conditions. Glutamate **(B,D)** is presented as both the 2 (C2 **(D)**) and 4 (C2 **(B)**) labeled carbon subpools. Lactate **(E)** is presented as the uniformly labeled ^13^C3-labeled lactate. Ribonucleic acids **(F)** are presented as the ^13^C positions 1 to 4 and ^13^C positions 3 to 5 subpools. Fatty acids **(G)** are presented as 2-carbon ^13^C-labeled palmitate and oleate. Error bars are mean ± SD, significant *P* values are indicated (*P ≤0.05, **P ≤0.01, ***P ≤0.001).

We compared the contribution of U^13^C glutamine to the same group of metabolites Figure 2A under control or acidosis (the relative changes are indicated in Figure 2, while the complete data sets, presented as ONCOisobolome matrix tables, are shown in Additional file 1: Tables S4 and S5). A similar media level of ^13^C^5^-labeled glutamate indicated equal availability of extracellular glutamine throughout the experiment (Figure 2B). Acidosis significantly increased the levels of total labeled ^13^C-CO_2_ produced from glutamine (Figure 2C) and partially labeled glutamate (Figure 2D), indicating increased utilization of glutamine by the TCA cycle. Furthermore, uniformly labeled lactate was decreased under acidosis (Figure 2E), consistent with both reduced lactate production and decreased malate shuttling, suggesting complete TCA metabolism. Acidosis also increased the ribonucleic acid labeling from glutamine, in particular ^13^C^3-5^ labeled ribose (Figure 2F). Glutamine-derived intermediates can label ribose via export from the mitochondria followed by entry into the non-oxidative branch of the PPP, so increased labeling of ribose by glutamine suggests an induction of the non-oxidative branch of the PPP. Finally, we observed no change in the labeled fatty acids under acidosis with the U^13^C glutamine tracer, suggesting similar rates of fatty acid synthesis (Figure 2G). This also indicates that there is no significant reduction carboxylation of glutamate, under acidosis alone, a notable difference from the cellular adaptations to hypoxia.

We then examined how acidosis affects palmitate metabolism using (U)^13^C palmitate for similar group of metabolites (Additional file 2: Figure S2A). The relative changes are displayed in Additional file 2: Figure S1, while the complete data sets are presented as ONCOisobolome matrix tables in Additional file 1: Tables S6 and S7) and found that acidosis reduced intracellular ^13^C-labeled palmitate (Additional file 2: Figure S1B), increased total labeled ^13^C-CO_2_ being produced from palmitate (Additional file 2: Figure S1C) and increased ^13^C^2^-labeled and ^13^C^4^-labeled glutamate (Additional file 2: Figure S1D). Collectively, these changes suggest that acidosis triggers an increase in the β-oxidization of palmitate to acetyl-CoA and subsequent feeding into the TCA cycle. Interestingly, there was a dramatic decrease in total ^13^C-labeled lactate levels (Additional file 2: Figure S1E), suggesting decreased malate shuttling. Ribose labeling from palmitate was also increased, in particular ^13^C^3-5^ labeled ribose (Additional file 2: Figure S1F). Finally, acidosis did not cause significant changes in labeled oleate from palmitate (Additional file 2: Figure S1G). The observed increase in β-oxidation would lead to both increased TCA cycle turnover and increased cellular NADPH generation, while the increase labeling of both ribose and 4-carbon-labeled glutamate suggested increased shuttling of TCA cycle intermediates to the cytosol, for use in either the non-oxidative PPP or recycling through glycolysis. Overall, our findings from the palmitate tracer indicate a pattern of metabolic reprogramming that is consistent with that seen for the glucose and glutamine tracers.

### The importance of glutaminolysis for metabolic adaptations under acidosis

**Figure 3:**
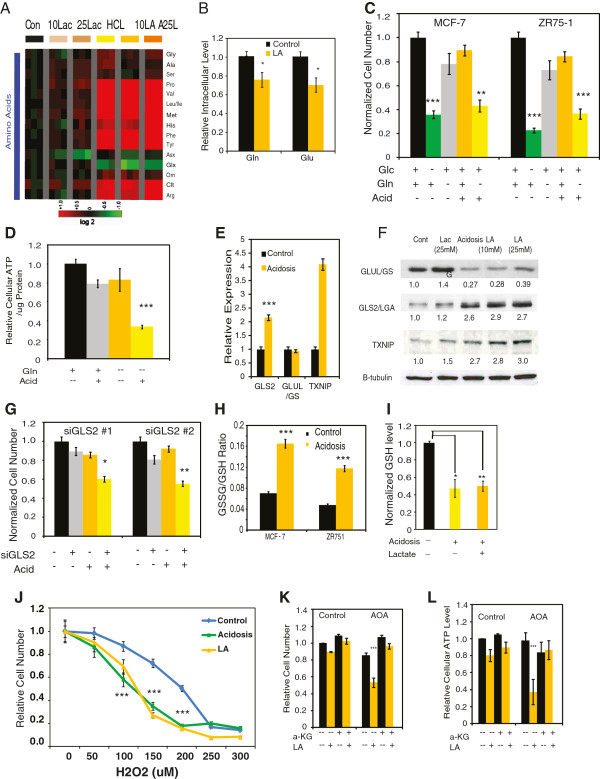
**Acidosis increases glutaminolysis and renders glutaminolysis essential. (A)** Heatmap shows the changes in the intracellular amino acids under acidosis (HCl), lactosis (10 and 25 mM Lac), or lactic acidosis (10 and 25 mM LA) (n = 3) conditions. **(B)** Normalized levels of intracellular glutamine (Gln) and glutamate (Glu) in MCF-7 cells under control or lactic acidosis conditions (n = 4). **(C)** Relative cell numbers as determined by trypan blue exclusion, of MCF-7 and ZR-75-1 cells after 72 h under the indicated media conditions. (n = 4) **(D)** Relative ATP levels under acidosis (Acid) with (+) or without (-) of glutamine (Gln) in media. **(E,F)** Relative changes of indicated mRNAs **(E)** and proteins **(F)** under indicated conditions. **(G)** Relative cell numbers of MCF-7 cells when transfected with control or two *GLS2* small interfering (si)RNAs under control and acidosis conditions. **(H)** Total glutathione (GSH) level of MCF-7 cells exposed to acidosis or lactic acidosis (n = 3). **(I)** Normalized glutathione disulfide (GSSG)/GSH ratios for MCF-7 and ZR-75-1 cells under control, acidosis or lactic acidosis conditions (n = 6). **(J)** Normalized cell numbers of MCF-7 cells under control, acidosis and lactic acidosis conditions when exposed to indicated level (uM) of H_2_O_2_ (n = 3). **(K,L)** Relative cell numbers (via trypan blue exclusion) **(K)** and cellular ATP level for MCF-7 **(L)** treated with vector or 0.2 mM amino-oxyacetate (AOA) for 72 h. The indicated samples were supplemented with 700 μm α-ketoglutarate (α-KG). Error bars are mean ± SEM, *P* values as indicated (*P ≤0.05, **P ≤0.001, ***P ≤0.0001).

Previous studies have indicated an increased reliance upon mitochondrial metabolism under acidosis [5,36], and the glutamine tracer experiments further support a major role for increased glutaminolysis in the increase in TCA cycle. To further examine how acidosis affects glutamine and other amino acids, we measured the intracellular levels of 15 amino acids in MCF-7 cells when exposed to 10 mM and 25 mM LA (pH 6.7) to represent the medium and upper range of tumor lactate [37] and acidity [13,34]. While both acidosis and lactic acidosis increased the levels of most amino acids (such as valine and leucine/isoleucine; Additional file 3: Figure S2A), they significantly reduced Glx (glutamine plus glutamate) (Figure 3A). When independent methods were used to resolve glutamine vs. glutamate in the Glx analysis, we found that acidosis reduced the intracellular levels of both glutamine and glutamate (Figure 3B). This depletion of glutamine and glutamate, in light of a general increase in most other amino acids, further support increased glutaminolysis under acidosis (Figure 2D).

However, given that acidosis has been shown to inhibit uptake of glucose [5], we wished to test how acidosis affects glutamine uptake by either consumption of glutamine in media (Additional file 3: Figure S2B) or cellular uptake of ^14^C-labeled glutamine (Additional file 3: Figure S2C). In both cases, we observed that acidosis increased both glutamine consumption and uptake. Therefore, the reduced glutamine/glutamate levels under acidosis are likely due to an increase in consumption (glutaminolysis) instead of reduced uptake. This represents an additional line of evidence that cells exposed to acidosis are increasingly reliant upon glutamine.

**Figure 4:**
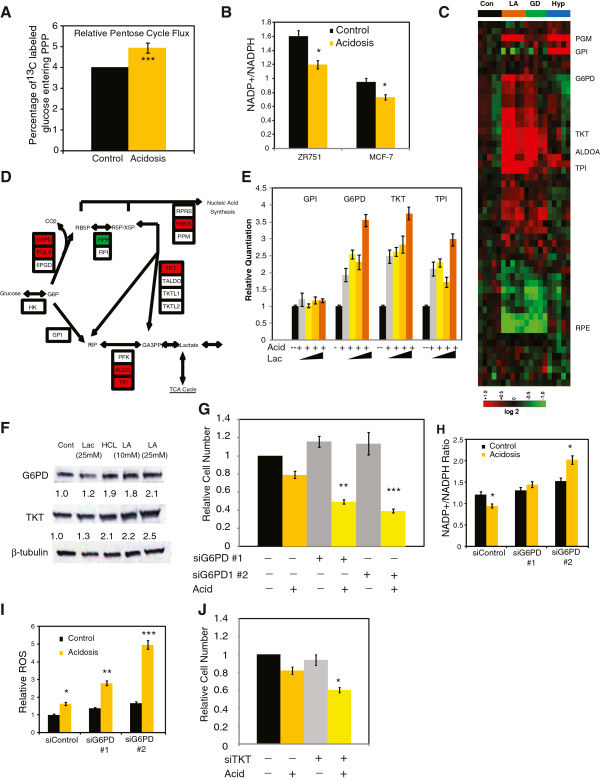
**Acidosis enhanced oxidative branch of pentose phosphate pathways (PPPs). (A)** Percentage of glucose that enters the PPP under control or acidosis conditions. **(B)** NADP+/nicotinamide adenine dinucleotide phosphate (NADPH) ratio for MCF-7 and ZR-75-1 cells under control or acidosis conditions (n = 6). **(C)** Heatmap indicates the transcriptional changes of genes listed by the Kyoto Encyclopedia of Genes and Genomes (KEGG) in the PPPs under hypoxia, lactic acidosis or glucose deprivation conditions. **(D)** Genes in PPP were induced (red) or repressed (green) by at least 1.7-fold under lactic acidosis. **(E)** The mRNA expression of *glucosephosphate isomerase (GPI), glucose-6-phosphate dehydrogenase (G6PD), transketolase (TKT), and triose phosphate isomerase (TPI)* under control or acidosis conditions with 0, 4, 10 and 25 mM lactate. **(F)** The protein levels of G6PD, TKT and β-tubulin in MCF-7 cells under acidosis, lactosis (25 mM), or lactic acidosis (10 mM and 25 mM pH 6.7) conditions with the relative changes (by densitometry) shown. **(G)** Relative cell numbers of MCF-7 cells that have been transfected with control or two small interfering (si)RNAs targeting *G6PD* under control or acidosis conditions. **(H)** Relative NADP+/NADPH ratios in MCF-7 cells, transfected with control or two G6PD-targeting siRNAs, under control or acidosis conditions (n = 4). **(I)** Relative ROS in MCF-7 cells transfected with control or two G6PD-targeting siRNAs, under control or acidosis conditions. **(J)** Relative cell numbers of MCF-7 cells transfected with control or *TKT*-targeting siRNA under control or acidosis conditions **(A)**. Error bars are mean ± SEM, *P* values as indicated (*P ≤0.05, **P ≤0.001, ***P ≤0.0001).

Next, we examined cellular phenotypes when cells were deprived of glutamine under acidosis. While MCF-7 and ZR75-1 cells were mostly glutamine-independent under neutral pH, glutamine deprivation under acidosis significantly decreased both cell numbers and ATP levels at either 4 or 24 h (Figure 3C,D, and Additional file 3: Figure S2B). The decrease in cell numbers associated with the combination of both acidosis and glutamine deprivation was comparable to the decrease under glucose deprivation, yet was largely absent when only acidosis or glutamine deprivation was present (Figure 4C). Figures 3C,D together suggest that at least one important role glutamine has under acidosis is ATP generation (bioenergetics). Glutamine can be used to generate ATP via anapleurosis, in which glutamine is sequentially converted to glutamate and then to α-KG to enter the TCA cycle.

Therefore, we next investigated how acidosis affected various enzymes involved in glutamine anapleurosis. Glutamine and glutamate are able to reciprocally interconvert via the activity of two enzymes: glutamine synthetase (GS, encoded by *GLUL*) and glutaminase (encoded by *GLS* and *GLS2*). We previously demonstrated that MCF-7 and other luminal breast cancer cells only expressed *GLS2*[38]. Both acidosis and lactic acidosis induced *GLS2* mRNA and protein (Figure 3E,F) levels while repressing the level of GS protein (Figure 3F). *TXNIP*, a gene previously shown to be induced under acidosis [36], served as a positive control. Such changes may explain the increased glutaminolysis and reduction in the intracellular glutamine under acidosis. The silencing of *GLS2* (Additional file 3: Figure S2D) considerably reduced cell numbers under acidosis (Figure 3G, Additional file 3: Figure S2E, F) in both MCF-7 and ZR-75-1 cells. Therefore, GLS2 induction and increased glutaminolysis were important for cell survival under acidosis.

A major role of glutamine and glutamate in cellular metabolism is, in combination with cysteine and glycine, the generation of glutathione for neutralizing reactive oxygen species. We therefore examined the glutathione status under acidosis and found that acidosis dramatically increased the GSSG (oxidized glutathione)/GSH (reduced glutathione) ratio and lowered the anti-stress capacity of cells after 24 h (Figure 3H). This increase in GSSG/GSH ratio was due to a simultaneous increase in GSSG levels coupled with a decrease in total glutathione levels (Figure 3I, Additional file 4: Figure S3A).

Both lactic acidosis and acidosis had a similar (approximately 40%) reduction in GSH levels (Figure 3I, Additional file 4: Figure S3A), suggesting that the effect is driven by acidosis. Also of note was the fact that cellular NADP+/NADPH and GSSG/GSH ratios, as well as total GSH (Additional file 4: Figure S3B-D) were also reduced, though not to the same degree, after 5 h of exposure to acidosis, similar to what was seen for cellular ATP levels (Additional file 3: Figure S2B).

These data suggest that these cellular adaptations to acidosis are progressive in nature and increase over the course of exposure to acidosis.

Consistent with compromised anti-stress capacity reflected in a higher GSSG/GSH ratio, both acidosis and lactic acidosis reduced the survival of MCF-7 cells under different levels of H_2_O_2_ (Figure 3J). To determine why total glutathione levels were decreased under acidosis, we examined the RNA levels of genes involved in the GSH synthesis [7]. We found that both subunits (*GCLC* and *GCLM*) of the enzyme glutamate-cysteine ligase, which catalyzes the first and rate-limiting step of GSH synthesis, were significantly downregulated under lactic acidosis (Additional file 5: Figure S4A). This decrease in *GCLC* and *GCLM* RNA levels was coupled with a decrease in NRF2 activity (Additional file 5: Figure S4B) under lactic acidosis, suggesting that acidosis (and/or lactic acidosis) decouples the reduced glutathione synthesis from glutaminolysis via inhibition of NRF2-regulated transcriptional activity. Since acidosis increases ROS levels within the cells, we wondered whether overexpression of NRF2 would reduce cellular ROS and mitigate the reduced viability under acidosis. Surprisingly, while NRF2 did lead to increased mRNA levels of *GCLC* and *GCLM* (Additional file 5: Figure S4C), it also caused a far greater decrease in cellular proliferation under acidosis (Additional file 5: Figure S4D). This decrease in cell numbers when *NRF2* is overexpressed under acidosis was correlated with a decrease in intracellular glutamine and glutamate (Additional file 5: Figure S4E), suggesting that redirection of glutamine and glutamate under acidosis is necessary for cell growth.

The redirection of glutamate away from GSH synthesis was quite striking, so we further examined the cellular requirement for glutamate under acidosis. Our stable isotope tracer data suggested that glutamine was increasingly redirected towards mitochondrial metabolism under acidosis (Figure 2D). The entry of glutamate into the TCA cycle requires the conversion of glutamate to α-KG, which occurs via either deamination or transamination. Glutamate deamination in non-neuronal cells is catalyzed by glutamate dehydrogenase 1 (GDH), encoded by *GLUD1*. Transamination, by contrast, is mediated by several classes of transaminases. Extracellular acidosis led to the specific downregulation of *GLUD1,* suggesting that glutamate deamination may not play a critical role in the cellular acidosis response (Additional file 5: Figure S4F) with *TXNIP* induction as positive control acidosis. Therefore, we tested the importance of glutamate transamination by determining how amino-oxyacetate (AOA), a chemical inhibitor of transamination, impacts cellular survival under acidosis. Cells treated with 0.2 mM AOA showed dramatically decreased cell numbers only under acidosis (Figure 3K), suggesting an important role for transamination under acidosis. We also found that membrane-permeable dimethyl α-KG significantly restored cell numbers (Figure 4K) and ATP levels (Figure 3L) of MCF-7 cells exposed to AOA under lactic acidosis. Similar synthetic lethality of AOA under acidosis and rescue by dimethyl α-KG was also observed for ZR-75-1 cells (Additional file 5: Figure S4G). Together, these results indicate that both acidosis redirects cellular glutamine to the TCA cycle for cellular bioenergetics (ATP generation), which leads to depletion of other glutamine-dependent metabolites, such as GSH.

### Redirection of glucose and the importance of NADPH to the cellular acidosis response

Collectively, the stable-isotope tracing experiments indicated that acidosis redirected glucose away from glycolysis and lactate production towards both the TCA cycle and the PPP. We therefore examined why cells would redirect glucose in this fashion, especially in light of the increased glutaminolysis and reduced GSH production. When the pentose-cycle flux was calculated based upon the ratio of ^13^C^1^-labeled and ^13^C^2^-labeled lactate, we found the percentage of glucose that entered the PPP increased from 4% to 5% under acidosis (Figure 4A). Since the extracellular glucose pool was 50% labeled, this change would correspond to an increase from 8% to 10% of total glucose entering the PPP. Since two NADPH are generated per single redirected glucose molecule, this 25% increase in PPP should generate considerably more NADPH. Indeed, we found that acidosis significantly increased the reductive capacity of cells, as indicated by a reduced NADP^+^/NADPH ratio, by 28% in MCF-7 cells and 39% in ZR-75-1 cells (Figure 4B). This decrease in the NADP+/NADPH ratio was generated by both a decrease in NADP + levels and an increase in NADPH levels (Additional file 6: Figure S5A).

To investigate the causes for increased PPP under acidosis, we examined the transcriptional response of PPP genes listed in the Kyoto Encyclopedia of Genes and Genomes (KEGG) in a previously reported microarray data [6] (Figure 4C,D). While the published data focused on lactic acidosis, the strong concordance reported between the acidosis and lactic acidosis responses [5] provides the rationale to use this data to investigate the acidosis response of MCF-7 cells. Analysis of the microarray data for all the KEGG-listed PPP genes indicated that lactic acidosis induced the expression of *G6PD*, *TKT* and triose phosphate isomerase (TPI) (Figure 4C,D). The induction of these genes under acidosis was further verified using real-time PCR in MCF-7 (Figure 4E) and ZR-75-1 cells (Additional file 6: Figure S5B). In addition to increased mRNA expression, we observed *G6PD* induction at the protein level under both acidosis and lactic acidosis (Figure 4F) and both MCF-7 and ZR-75-1 cells had increased intracellular G6PD activity under acidosis (Additional file 6: Figure S5C). Since G6PD encodes the first and rate-limiting step of the oxidative branch of the PPP the increased G6PD RNA, protein and activity levels may explain the increase in the oxidative PPP activity under acidosis.

To test this possibility, we determined the impact of silencing of G6PD on cell numbers under control or acidosis conditions. While acidosis alone reduced cell numbers by approximately 20%, the silencing of G6PD by two separate siRNAs (Additional file 6: Figure S5D) further reduced cell numbers by approximately 60%, as assayed after 96 h, by either trypan blue exclusion or PI incorporation, for both MCF-7 and ZR-75-1 cells (Figure 4G, Additional file 6: Figure S5E-G). Similarly, a PPP inhibitor, 6-aminonicotinamide (6-AM), also exhibited similar acidosis-specific reduced survival (Additional file 6: Figure S5H). In contrast, 2-deoxyglucose (2-DG), an inhibitor of glycolysis, improved survival under acidosis (Additional file 6: Figure S5H). Collectively, these data suggest that G6PD and the PPP are critical for cellular survival under acidosis.

**Figure 5:**
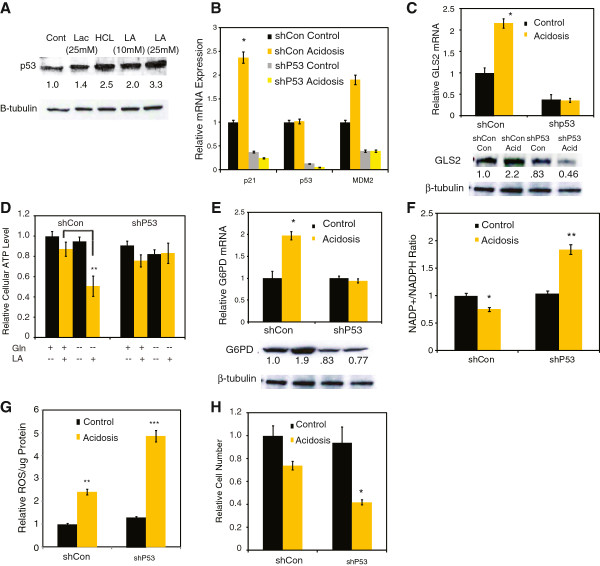
**Regulation of the metabolic response to acidosis by p53. (A)** Protein levels of p53 under control, lactosis, acidosis (HCl) and lactic acidosis (LA). **(B-D)** Relative mRNA expression of p21, p53 and MDM2 genes in the shControl or shP53 MCF-7 cells under control or acidosis conditions. **(C)** Relative mRNA expression and protein levels of GLS2 in shControl or shP53 MCF-7 cells under control or acidosis conditions. **(D)** Relative ATP levels in shControl or shp53 cells under lactic acidosis (LA) with (+) or without (-) glutamine. **(E)** Relative mRNA expression and protein levels of glucose-6-phosphate dehydrogenase (G6PD) in shControl or shP53 MCF-7 cells under control or acidosis conditions. **(F-H)** Relative NADP+/nicotinamide adenine dinucleotide phosphate (NADPH) ratio **(F)**, normalized ROS levels **(G)**, and normalized cell numbers **(H)** in shControl or shP53 MCF-7 cells under control or acidosis conditions. Error bars are mean ± SEM, *P* values as indicated (*P ≤0.05, **P ≤0.001, ***P ≤0.0001).

We then investigated why G6PD is critical for cellular survival under acidosis. The oxidative branch of the PPP is important for generating NADPH, an important reducing equivalent for lipogenesis and neutralizing ROS. Since there is no increase in fatty acid synthesis (Figure 1G), NADPH generated by G6PD may be required for ROS neutralization. Silencing G6PD further mitigated the decrease in NADP+/NADPH (Figure 5H) and increased the levels of ROS under acidosis (Figure 4I). These data suggest that G6PD is essential for the generation of NADPH to neutralize the increased ROS under acidosis.

Finally, our stable isotope tracer data for both glutamine and palmitate (Figure 2F, and Additional file 2: Figure S1F) also indicated that acidosis induced the non-oxidative branch of the PPP. The non-oxidative branch of the PPP, which reversibly returns PPP intermediates back to glycolysis, is primarily mediated by two enzymes, TKT1 and transaldolase. To test whether induction of the non-oxidative branch of the PPP was critical to the cellular response to acidosis, we assessed cell proliferation under acidosis after siRNA silencing of *TKT1*. While not as severe an effect as observed for G6PD, the combined silencing of *TKT* and exposure to acidosis reduced cell proliferation by approximately 50% after 72 h (Figure 4J). This suggests that while glucose is redirected to the PPP, cells also require glucose-based PPP intermediates to be redirected back to glycolysis, which may explain the dramatic decrease in glucose labeling of ribose sugars.

### The role of p53 in the coordination of the metabolic response to acidosis

Next, we wanted to identify potential regulators of the observed metabolic reprogramming under acidosis. Many studies have indicated that both enhanced PPP and increased mitochondrial metabolism can be triggered by the activation of the tumor suppressor p53 [39]. Furthermore, there is a highly positive correlation between the lactic acidosis response elicited in MCF-7 cells and the p53 pathways in the breast tumor datasets [8]. Acidosis also decreased malate flux from the TCA cycle (Figure 1E, Additional file 2: Figure S1E), a process that was recently shown to be inhibited by p53 [40]. Based upon these previous findings, we hypothesized that p53 may play a role in the metabolic response to acidosis. Consistent with that hypothesis, we found that acidosis increased p53 protein levels (Figure 5A) and induced the expression of *p21* and *MDM2*, two well-known p53 target genes (Figure 5B). Therefore, we tested whether p53 may play a role in the metabolic response of acidosis by comparing isogenic groups of MCF-7 cells with or without p53 by stably expressed small hairpin (sh)RNAs (Additional file 7: Figure S6A) [22]. First, we noted that the induction of GLS2 mRNA and protein under acidosis was abolished in the p53-deficient cells (Figure 5C). The reduced ATP during glutamine deprivation and lactic acidosis was also largely abolished when p53 was inhibited (Figure 5D). These results indicate the GLS2 induction and altered glutamine metabolism under acidosis is a p53-dependent process.

**Figure 6:**
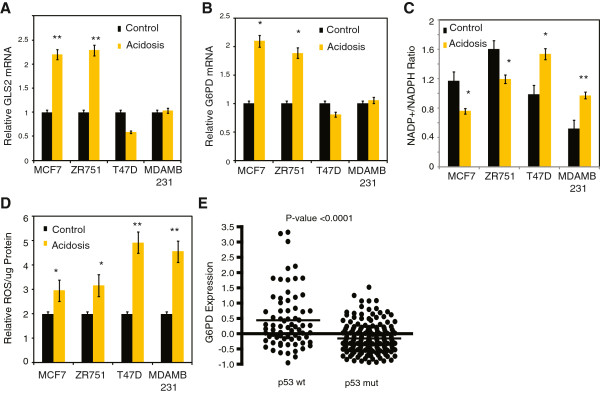
**Glucose-6-phosphate dehydrogenase (G6PD) and p53 status are correlated *in vivo*. (A)** Relative mRNA expression of GLS2 in the indicated cell lines under control or acidosis conditions. **(B)** Relative mRNA expression of G6PD in the indicated cell lines under control or acidosis conditions. **(C,D)** Relative NADP+/nicotinamide adenine dinucleotide phosphate (NADPH) ratio **(C)** and normalized ROS levels **(D)** in the indicated cell lines under control or acidosis conditions. **(E)** Relative expression level of *G6PD* mRNA in among groups of breast tumors with wild-type or mutant p53.

Similarly, we found that either the genetic or chemical silencing of p53 abolished the induction of G6PD mRNA and protein under acidosis (Figure 5E, Additional file 7: Figure S6B,C). Consistent with the role of p53 in the enhancement of the PPP, the silencing of p53 also significantly increased both NADP+/NADPH ratio (Figure 5F) and ROS (Figure 2G), similar to the silencing of G6PD (Figure 4I,J). p53 mediation of these metabolic adaptations to acidosis appears to play an important role in cellular proliferation, as p53-deficient cells also have dramatically reduced cellular proliferation under acidosis (Figure 5H). Furthermore, acidosis failed to induce *GLS2* and *G6PD* in several breast cancer cell lines with mutated p53 (T47D and MDA-MD-231) (Figure 6A,B, Additional file 7: Figure S6D). Finally, two cell lines with mutated p53 also showed decreases in the reductive capacity (increased NADP+/NADPH ratio) (Figure 6C) and higher levels of ROS (Figure 6D) under acidosis. Therefore, p53 activation under acidosis is responsible for the induction of both glutaminolysis and the oxidative branch of the PPP to mitigate the oxidative stress under acidosis.

p53 is known to transcriptionally regulate a number of genes involved in cellular metabolism. However, given the lack of induction of *GLS2* under acidosis when p53 was genetically inhibited, we wanted to examine loss of GLS2 induction could account for the lack of adaptive response in the cells without p53. To test this possibility, we forced the expression of GLS2 by transfecting GLS2 and empty vectors in the control and p53-knockdown MCF-7 cell lines. When these cells were placed under control or acidosis conditions, we found that expression of GLS2 in the p53-deficient MCF-7 cells partially rescues the increased ROS, increased NADP+/NADPH ratio and mitigates the decrease in cell numbers seen when p53 is genetically inhibited under acidosis (Additional file 7: Figure S6E-G). These data strongly suggest that the p53-dependent GLS2 induction play an important role in the anti-stress capacity under acidosis. However, other p53 targets may also play a role in the adaptive cellular response to extracellular acidosis.

To extend the relationship between p53 and *G6PD*, we examined their relationship *in vivo* using a breast cancer dataset with known p53 status [41]. Tumors with wild-type p53 (n = 72) had significantly higher *G6PD* expression than p53 mutant tumors (n = 179) (Figure 6E), indicating that the connection between p53 activity and G6PD expression extends beyond cell lines to human tumors as well.

## Discussion

Relatively little is known about the cellular metabolic response to acidosis found in most solid tumors. Here, we employ isotope tracer experiments to make several important observations on how acidosis affects glucose, glutamine and palmitate metabolism. Besides confirming previous observations of reduced glycolysis and lactate production [6,7], these experiments revealed that extracellular acidosis increased oxidative PPP (Figures 1 and 4), glutaminolysis (Figure 2) and β-oxidation of fatty acids (Additional file 2: Figure S1). Interestingly, acidosis also abolished most of the conversion of glucose to ribose for ribonucleotide synthesis via the PPP. Since the decrease in ribose conversion (approximately 90%) far exceeds the reduction in proliferation (approximately 30% in 3 days), there is possibly enzymatic inhibition or reversed metabolic flow in the non-oxidative PPP under acidosis. Such increased oxidative PPP and reduced ribose production under acidosis is reminiscent of the metabolic patterns of the quiescent fibroblasts [42]. These metabolic adaptions allow cells to cope with the demand for bioenergetic needs (ATP) and reducing equivalents (NADPH) necessary for survival, while reducing the biosynthetic requirements (that is, ribonucleotides) of cellular proliferation. These changes are also consistent with the starvation response [6] and increased autophagy [16,43] under acidosis. The diversion of glucose to the PPP may further increase the need for TCA metabolites, which can be supplied from the increased glutaminolysis and fatty acid β-oxidation. A similar increased need for NADPH and glutaminolysis has been observed for other metabolic stresses, such as hypoxia [44], glucose deprivation [45] and matrix detachment [46]. Therefore, these shared metabolic adaptations may underlie common mechanisms of cellular responses to various tumor environmental stresses within solid tumors.

We also identified a critical role for p53 in coordinating various metabolic responses to acidosis. The increase in mitochondria function (for ATP generation) and the oxidative branch of the PPP (for NADPH to neutralize ROS) are both known functions of p53 in (1) promoting catabolic pathways to maintain energy production during nutrient starvation; (2) ameliorating oxidative stress; and (3) inhibiting cell growth and cell cycle progression. Acidosis also reduces glycolysis [5,6], which is another prominent metabolic feature downstream of p53 through the induction of *TP53*-inducible glycolysis and apoptosis regulator (*TIGAR*) [47] and downregulation of glucose transporters [48] and phosphoglycerate mutase [49]. The induction of GLS2 and increased glutaminolysis under acidosis, while novel, is also consistent with the function of p53 to promote ATP production and antioxidant function [24,50]. p53 has also been shown to downregulate NRF-2-mediated transcription [51], suggesting that the decrease in GSH synthesis under acidosis, due to downregulation of GCLC and GCLM, is also consistent with increased p53 activity under lactic acidosis. Therefore, the response to extracellular acidosis may represent a novel tumor suppressor function of p53. Furthermore, the dependence of the acidosis response on p53 may explain the higher degree of lactic acidosis transcriptional responses in breast tumors with wild-type p53 [5,8] even though these tumors, when compared tumors with mutant p53, have lower levels of lactic acidosis, glycolysis and hypoxia [8].

G6PD encodes the first and rate-limiting enzyme of the oxidative PPP. Therefore, the increase in both *G6PD* expression and activity under acidosis (Figure 5) is important for the enhanced oxidative PPP activities. Several mechanisms are likely to account for the increase in G6PD activity under acidosis by p53. We noted a p53-dependent transcriptional induction of G6PD, consistent with a previous report of *G6PD* as transcriptional target of p53 [52]. The increased NADP+/NADPH under acidosis may also activate G6PD allosterically [45]. Cytoplasmic p53 has been reported to inhibit G6PD protein directly through protein-protein interaction [40], so it is possible that acidosis may reduce the interaction by lowering cytoplasmic pools of p53 or pH-dependent conformational changes. While the contribution of individual factors remains to be determined, the induction of G6PD is likely an important adaptation under acidosis when cells become susceptible to genetic and chemical inhibition of G6PD.

While this study provides many new insights of cellular metabolism under acidosis, many aspects of the metabolic adaptations of cells to acidosis still remain unknown. For example, we still do not understand how acidosis enhances the oxidative PPP while abolishing ribonucleotide production via the non-oxidative PPP. Such dissociation between these two branches of the PPP has been previously described for Ras-driven pancreatic cancers [53] and may save the cells from wasting glucose metabolites in nucleotide biosynthesis given the lack of proliferation under acidosis. However, we also observe an increase in the flow of TCA cycle metabolites, via the non-oxidative branch of the TCA cycle, into ribose labeling. Thus, it is clear that some of the TCA cycle components are being used for non-bioenergetic purposes under acidosis, though the mechanism by which TCA metabolites, potentially including increased export of citrate and malate, enter the cytosol remains unclear. However, glutamine and palmitate contribute only a low level of ribose synthesis, which would appear to be insufficient to compensate for the loss of glucose labeling of ribose. This leads us to conclude that a combination of metabolites, potentially including glycerol from the fetal bovine serum and potentially circulating triglycerides in humans, not just glutamine or palmitate, contribute to the labeling of ribose under extracellular acidosis.

Furthermore, while we investigated the transcriptional response of G6PD and GLS2, there are likely to be additional regulators of the acidosis response, at either the transcriptional, translational or post-translational level. For example, p53-responsive *TIGAR* may also play a role in the shunting of glucose to PPP for NADPH [39] under acidosis. The roles of additional regulators and metabolic flow will be studied in detail in the future to gain a more complete understanding of the metabolic response to acidosis.

**Figure 7:**
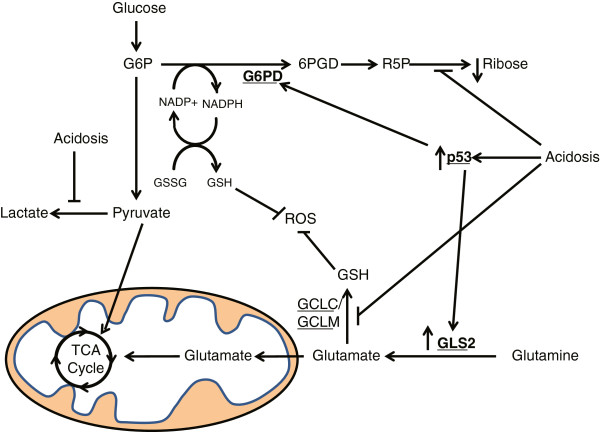
**Overview of the metabolic reprogramming mediated by p53 under acidosis conditions.** Glucose-6-phosphate dehydrogenase (G6PD) is induced under acidosis by p53, which increases nicotinamide adenine dinucleotide phosphate (NADPH) from the oxidative PPP appears to help cells tolerate the increased ROS stresses and reduced novel GSH synthesis under acidosis conditions. Acidosis also dramatically reduces the novel RNA ribose synthesis via the PPP. Furthermore, the activation of p53 under acidosis contributes to increased glutaminolysis by inducing *GLS2*, leading to increased glutamate generation, which is then converted to α-ketoglutarate (α-KG) and enters the tricarboxylic acid cycle (TCA cycle). Enzymes are underlined and in bold.

The *in vivo* relevance of many of our observations on the regulation of metabolic reprogramming has been independently confirmed in other systems and disease settings. For example, metabolic acidosis is noted to increase glutaminolysis in intact nephron and rodent models, possibly mediated by increased glutaminase (*GLS*) mRNA stability [54]. Previous studies have noted the regulation of liver-type glutaminase (GLS2), glutamine metabolism and antioxidative capacity by p53 in breast cancers [24,50]. The strong positive correlation of G6PD expression, the acidosis transcriptional response and p53 status further support the role of p53 in inducing G6PD and other cellular acidosis responses (Figure 7). It is also worth noting that the G6PD locus is locally amplified in breast and other tumors in Tumorscape [55]. Such hardwired DNA amplification may confer a robust PPP and a selective advantage within the tumor microenvironmental stresses.

Tumor acidosis is often associated with metastasis and resistance to cancer therapeutics in patients [6,7]. Interestingly, the two major changes we observed here in cancer cells under acidosis, elevated levels of G6PD and increased PPP, both of which show the importance of the regeneration of reducing equivalents to cancer proliferation, are also both also associated with brain metastasis [56,57] and drug resistance [58,59]. Additionally, extracellular acidosis forces breast cancer cells to engage in a series of dysregulated metabolic adaptations in order to adapt to the changing extracellular environment. Since increased oxidative stress, as occurs in response to acidosis, is associated with metastasis and drug resistance, it seems logical for cells to reprogram their metabolism to cope with this type of increased stress. The observed metabolic changes under acidosis could also reflect a novel tumor suppressive role for p53; while p53 promotes TCA cycle metabolism, it also induces the PPP to mitigate the increased ROS and DNA instability from TCA metabolism, which ultimately impedes tumor progression. Together, the data presented here provide several novel insights into both the short-term metabolic adaptations of cancer cells under acidosis, as well as the long-term potential impact of acidosis to exert selective pressures upon the somatic mutations within human tumors.

## Conclusions

Taken together, our metabolomic analysis of the acidosis responses indicates a significant reprogramming of cellular metabolism toward the oxidative PPP and glutaminolysis through the induction of *G6PD* and *GLS2*, respectively (Figure 7). The increase in the NADPH from the oxidative PPP appears to be required to help cells to better tolerate the increased ROS stress and reduced novel GSH synthesis under acidosis. Furthermore, p53 plays an important role in coordinating the metabolic response to acidosis by inducing *G6PD* (redirection to the PPP) and *GLS2* (increased glutaminolysis). Such metabolic adaptations under acidosis may render cancer cells susceptible to inhibition of glutamate metabolism and/or NADPH generation.

## Abbreviations

6-AN: 6-aminonicotinamide; AOA: amino-oxyacetate; G6PD: glucose-6-phosphate dehydrogenase; GLS2: glutaminase 2; GSH: glutathione; LA: lactic acidosis; NADPH: nicotinamide adenine dinucleotide phosphate; PCR: polymerase chain reaction; PPP: pentose phosphate pathway; ROS: reactive oxygen species; TCA: tricarboxylic acid cycle; TKT1: transketolase 1.

## Competing interests

The authors declare they have no competing interests.

## Authors’ contributions

GML, XT, JL-YC, JW, C-KCD, CS, and H-NK designed and performed cell culture experiments, siRNA silencing, and microarray analysis and mRNA quantification. OI, XT, JL-YC, JW, and CBN prepared samples designed and performed the amino-acid profiling experiments. GML, MMK and LGB prepared samples, designed and performed the stable-isotope tracer experiments. C-CKD and H-NK performed the GLS2 overexpression experiments. J-TC designed the overall experimental focus, analyzed data and supervised all experiments. All authors read and approved the manuscript.

## Supplementary Material

Additional file 1: Table S1 Listing of all primers and small interfering (si)RNAs used in this manuscript. **Table S2-S7**: Oncoisobolome and EZTop tables containing all relative and absolute measurements for all metabolites profiled in the glucose (**Tables S2** and **S3**), glutamine (**Tables S4** and **S5**) and palmitate (**Tables S6** and **S7**) tracer studies. Metabolic profiles of MCF-7 cells in response to control (pH 7.4) or acidic (pH 6.7) conditions after 24 h of culture were obtained via SiDMAP analysis using [1,2-^13^C_2_]-d-glucose tracer, [U-^13^C_2_]-d-glutamine tracer, and [1,2-^13^C_2_]-palmitate tracer. Measured metabolites are as indicated, with identities determined and listed via M_n_/Σm: isotopomer/^13^C labeled fraction as SUM(*m*_1_ + *m*_2_ + .. + *m*_n_). Σ*m*_n_: molar enrichment (ME) ^13^C content as SUM(1 × *m*_1_ + 2 × *m*_2_ + .. + *n* × *m*_n_) (Lee *et al*.) (n = 4). Error bars are mean ± SD, *P* values as indicated (*P ≤0.05, **P ≤0.001, ***P ≤0.0001). Click here for file

Additional file 2: Figure S1 The use of ^13^C palmitate isotope tracer to analyze glutamine metabolism under acidosis. **(A)** Schematic graph indicating the measured metabolites (and corresponding panels) resulting from the uniformly ^13^C labeled palmitate tracer under control or acidosis conditions. The relevant substrate tracer is indicated in green, ^13^C labeled carbons are indicated in red (normal carbon atoms are black). **(B-G)**. Relative ^13^C enrichment in the palmitate **(B)**, CO2 **(C)**, glutamate **(D)**, lactate **(E)**, ribonucleic acids **(F)** and oleate **(G)** under control or acidosis conditions. Glutamate **(D)** is presented as both the 2 (C2 (E)) and 4 (C4 (B)) labeled carbon subpools. Lactate **(E)** is presented as the total ^13^C-labeled lactate pool. Ribonucleic acids (F) are presented as the ^13^C positions 1 to 4 subpool. Fatty acids **(B,G)** are presented as 2-carbon ^13^C-labeled palmitate **(B)** and oleate **(G)**. Error bars are mean ± SD, significant *P* values are indicated (*P ≤0.05, **P ≤0.01, ***P ≤0.001). Click here for file

Additional file 3: Figure S2 Essential role of glutaminolysis under acidosis. **(A)** The intracellular levels of Val and Leu/Ile under indicated conditions of acidosis or lactic acidosis conditions (n = 3). **(B)** Normalized cellular ATP levels in MCF-7 cells under control or acidosis conditions after 4 h. **(C)** Measurements of glutamine in cell culture media at 5 and 24 h after exposure to acidosis. **(D)**^14^C-glutamine levels in cell pellets under control or acidosis conditions in MCF-7 cells at 1 h and 12 h. **(E)** Levels of the indicated proteins in the glutamine/glutamate metabolism pathways after the gene silencing by respective small interfering (si)RNAs. **(F,G)** Relative cell numbers (as a ratio of acidosis/control) of MCF-7 **(F)** and ZR-75-1 **(G)**, determined by propidium iodide staining, when the indicated genes were silenced under normal or acidosis conditions (n = 3). Error bars are mean ± SD, significant *P* values are indicated (*P ≤0.05, **P ≤0.01, ***P ≤0.001). Click here for file

Additional file 4: Figure S3 Effects of acidosis on glutathione (GSH)/glutathione disulfide (GSSG) and NADP+/nicotinamide adenine dinucleotide phosphate (NADPH) after 5 h of exposure **(A)** Normalized total GSH and GSSG levels for MCF-7 and ZR-75-1 cells under control or acidosis conditions (pH 6.7). **(B-D)** NADP/NADPH ratio, GSSG/GSH ratio, normalized total GSH levels of MCF-7 cells after 5 h of either control or acidosis conditions. Error bars are mean ± SD, significant *P* values are indicated (*P ≤0.05, **P ≤0.01, ***P ≤0.001). Click here for file

Additional file 5: Figure S4 Acidosis reduced nuclear factor erythroid 2-related factor 2 (NRF2) activities and increased levels of ROS. **(A)** Relative mRNA abundance, determined by microarray and quantitative real-time PCR (qPCR), for the indicated genes under control or lactic acidosis conditions. **(B)** Relative NRF2 activity, as determined by luciferase reporter, for MCF-7 cells exposed to control or lactic acidosis conditions. **(C)** Relative mRNA levels of the indicated genes, after green fluorescent protein (GFP) or NRF2 overexpression, as determined by qPCR. **(D)** Relative cell numbers 48 h after the expression of GFP or NRF2 in MCF-7 cells under control or acidosis conditions. **(E)** Intracellular normalized levels of glutamine and glutamate in MCF-7 cells that have been transfected with GFP or NRF2 expression constructs. **(F)** Relative transcript abundance, determined by microarray and qPCR, for the indicated genes under control, acidosis (qPCR only) or lactic acidosis conditions. **(G)** Relative cell numbers for ZR-75-1 cells treated with 0.2 mM amino-oxyacetate (AOA) or under control or acidosis conditions. Indicated cells are also supplemented with 700 uM dimethyl α-ketoglutarate (α-KG) (n = 4). Error bars are mean ± SD, significant *P* values are indicated (*P ≤0.05, **P ≤0.01, ***P ≤0.001). Click here for file

Additional file 6: Figure S5 The effects of acidosis on the expression of genes that encode proteins in the pentose phosphate pathways (PPPs). **(A)** Normalized NADP + and nicotinamide adenine dinucleotide phosphate (NADPH) levels in MCF-7 and ZR-75-1 cells under control and acidosis conditions. **(B)** The acidosis-induced change of mRNA expression for the indicated genes in MCF-7 and ZR-75-1 cells. **(C)** Relative glucose-6-phosphate dehydrogenase (G6PD) activity in MCF-7 and ZR-75-1 cells under control or acidosis conditions. **(D)** Protein levels of G6PD and transketolase 1 (TKT1) in MCF-7 cells transfected by control (siControl), two small interfering (si)RNAs targeting G6PD (siG6PD), or siRNA targeting TKT1 (siTKT1). **(E)** Relative cell numbers of ZR-75-1 cells transfected with control or siRNA targeting G6PD under control or acidosis conditions. **(F,G)** The change in cell numbers of MCF-7 **(F)** and ZR-75-1 **(G)** cells, under acidosis, treated with the indicated siRNAs determined by propidium iodide staining. **(H)** Relative cell numbers of MCF-7 cells that have been treated with dimethylsulfoxide (DMSO), 2-deoxyglucose (2-DG) or 6-aminonicotinamide (6-AM) under control or acidosis conditions (n = 4). Error bars are mean ± SD, significant *P* values are indicated (*P ≤0.05, **P ≤0.01, ***P ≤0.001). Click here for file

Additional file 7: Figure S6 The role of p53 in the acidosis response. **(A)** Protein levels of p53 in MCF-7 cells expressing shp53 or shControl. **(B)** Glucose-6-phosphate dehydrogenase (G6PD) activity in MCF-7 cells expressing shCon or shP53 exposed to either control or acidosis conditions. **(C)** G6PD activity in the indicated cell lines exposed to either control or acidosis conditions. **(D)** mRNA expression of the indicated genes exposed to either control or pifithrin-α under normal or acidosis conditions. **(E-G)** normalized ROS levels **(E)**, NADP+/nicotinamide adenine dinucleotide phosphate (NADPH) ratio **(F)** and normalized cell numbers **(G)** in the shControl or shP53 MCF-7 cells when they were transfected with either GLS2 or empty expression vector, under control or acidosis conditions. Error bars are mean ± SEM, *P* values as indicated (*P ≤0.05, **P ≤0.001, ***P ≤0.0001). Click here for file
