# Supplementary material for: Acidosis induces reprogramming of cellular metabolism to mitigate oxidative stress
Source: Cancer Metab. 2013 Dec 23;1:23. doi: 10.1186/2049-3002-1-23 (PMC4178214; doi:10.1186/2049-3002-1-23)
Supplement: Additional file 1: Table S1 — Listing of all primers and small interfering (si)RNAs used in this manuscript. Table S2-S7: Oncoisobolome and EZTop tables containing all relative and absolute measurements for all metabolites profiled in the glucose (Tables S2 and S3), glutamine (Tables S4 and S5) and palmitate (Tables S6 and S7) tracer studies. Metabolic profiles of MCF-7 cells in response to control (pH 7.4) or acidic (pH 6.7) conditions after 24 h of culture were obtained via SiDMAP analysis using [1,2-13C2]-d-glucose tracer, [U-13C2]-d-glutamine tracer, and [1,2-13C2]-palmitate tracer. Measured metabolites are as indicated, with identities determined and listed via Mn/Σm: isotopomer/13C labeled fraction as SUM(m1 + m2 + .. + mn). Σmn: molar enrichment (ME) 13C content as SUM(1 × m1 + 2 × m2 + .. + n × mn) (Lee et al.) (n = 4). Error bars are mean ± SD, P values as indicated (*P ≤0.05, **P ≤0.001, ***P ≤0.0001). [file 2049-3002-1-23-S1.zip › 1313128875104571_additional file 1_Table S1.pdf]

Gene	Primer F	Primer R	
B-actin	CTCTTCCAGCCTTCCTTCCT	AGCACTGTGTTGGCGTACAG	
TXNIP	CTGGCGTAAGCTTTTCAAGG	AGTGCACAAAGGGGAAACAC	
GCLC	ATCCTCCAGTTCCTGCACAT	GGGTAGGATGGTTTGGGTTT	
GCLM	TCAGTCCTTGGAGTTGCACA	ACACAGCAGGAGGCAAGATT	
NRF2	GCGACGGAAAGAGTATGAGC	GTTGGCAGATCCACTGGTTT	
GOT1	GGCCATTCGCTATTTTGTGT	GACCAAGTAATCCGCACGAT	
GOT2	AATGTTTGCCTCTGCCAATC	AGGGGAGGGTTGGAATACAT	
GPT1	GTGTCATCAACCCTGGCAAC	AATGAGTGGAACTGCGAACC	
GPT2	CGCCATCCAGGTGAATTACT	CCTCATCAGCCAGGAGAAAG	
GLUD1	GAGTCTCTCCTTCCCCATCC	GCACATCAACCACTGCACAC	
GLUL	CCTGCTTGTATGCTGGAGTC	GATCTCCCATGCTGATTCCT	
GS	GCTGTGCTCCATTGAAGTGA	GCAAACTGCCCTGAGAAGTC	
siRNAs	Ambion Catalog #	Ambion siRNA ID#	
GLUL	4390824	s421	
GLS2	4392420	s223734	
GOT1	4392420	s5954	
GOT2 #1	4392420	s5957	
GOT2 #2	4392420	s5959	
GPT1	4392420	s6103	
GPT2	4392420	s39366	
GLUD1	4390824	s15	

Table S1
