# Supplementary material for: Acidosis induces reprogramming of cellular metabolism to mitigate oxidative stress
Source: Cancer Metab. 2013 Dec 23;1:23. doi: 10.1186/2049-3002-1-23 (PMC4178214; doi:10.1186/2049-3002-1-23)
Supplement: Additional file 1: Table S1 — Listing of all primers and small interfering (si)RNAs used in this manuscript. Table S2-S7: Oncoisobolome and EZTop tables containing all relative and absolute measurements for all metabolites profiled in the glucose (Tables S2 and S3), glutamine (Tables S4 and S5) and palmitate (Tables S6 and S7) tracer studies. Metabolic profiles of MCF-7 cells in response to control (pH 7.4) or acidic (pH 6.7) conditions after 24 h of culture were obtained via SiDMAP analysis using [1,2-13C2]-d-glucose tracer, [U-13C2]-d-glutamine tracer, and [1,2-13C2]-palmitate tracer. Measured metabolites are as indicated, with identities determined and listed via Mn/Σm: isotopomer/13C labeled fraction as SUM(m1 + m2 + .. + mn). Σmn: molar enrichment (ME) 13C content as SUM(1 × m1 + 2 × m2 + .. + n × mn) (Lee et al.) (n = 4). Error bars are mean ± SD, P values as indicated (*P ≤0.05, **P ≤0.001, ***P ≤0.0001). [file 2049-3002-1-23-S1.zip › 1313128875104571_additional file 3_Table S3.pdf]

MCF-7	Glucose Tracer	Relative Measurements	pH		Statistical Tests			
**Metabolite**	**Summary**	**Isotopomer Fragment Dimension**	**7.40**	**6.70**	R square	Correlation	T test 1_tail	F test
**Complete Tracer Oxidation**		**13CO2 production**	100.0	106.4	1	1	0.5	1
**Lactate (m/z 328) (Media-CAS: 50-21-5)**	**Total 13C Lactate Pool**	**13C-labeled fraction (Σm)**	100.0	89.1	1	-1	0.165251444	0.675991
	**Pentose Cycle-derived Lactate**	**13C-m1 (m/z328) (m1/Σm)**	100.0	120.3	1	1	0.308255491	0.388857
	**Glycolysis-Derived Lactate**	**13C-m2 (m/z328) (m2/Σm)**	100.0	97.2	1	-1	0.18299327	0.525097
	**Lactate-Derived from glycolysis and labeled-TCA substrates**	**13C-m3 (m/z328) (m3/Σm)**	100.0	102.4	1	1	0.322441069	0.456801
	**Percentage of Lactate which is Pentose cycle-derived**	**Pentose cycle-glycolysis (m1/m2)**	100.0	122.6	1	1	0.301142994	0.351364
**Glutamate (c2-c5:m/z 198) (media-CAS:617-65-2)**	**13C Glutamate labeled by the tracer substrate via the TCA cycle**	**13C-labeled fraction (m/z198) (Σm)**	100.0	93.7	1	-1	0.146466419	0.989975
	**13C Glutamate labeled fraction produced by pyruvate dehydrogenase and citrate formation from tracer-derived acetyl-CoA and lost via glutamine-ketoglutaric aminotransferase from the TCA cycle**	**13C-m2 (m/z198) (m2/Σm)**	100.0	142.5	1	1	0.275131409	0.190305
	**13C Glutamate-labeled fraction produced via tracer derived acetyl-CoA and tracer-labeled oxaloacetate, then lost via glutamate transaminase II when the cycle is cut short.**	**13C-m4 (m/z198) (m4/Σm)**	100.0	118.4	1	1	0.315834356	0.4262
**Palmitate (C:16) (Pellet - CAS: 57-10-3)**	**13C Palmitate labeled by the substrate via labeled acetly-CoA**	**13C-labeled fraction (m/z 270) (Σm)**	100.0	74.8	1	-1	0.210100463	0.316667
	**13C Palmitate labeled by the substrate via tracer-derived acetly-CoA, with only 2 carbons labeled to reflect novel synthesis via the tracer substrate**	**Chain Elongation (m/z270) (m2/Σm)**	100.0	93.4	1	-1	0.146522204	0.980413
	**Percentage of total13C Palmitate labeled by the substratewhich represents newly synthesized palmitate**	**Fraction of New Synthesis (FNS) (% of Total)**	100.0	71.4	1	-1	0.2148129	0.280303
	**13C Acetyl CoA labeled by the substrate**	**Ace-CoA (% of Total)**	100.0	85.6	1	-1	0.182059201	0.5325
**Oleate (C18-1) (Pellet - CAS:112-80-1)**	**13C Oleate labeled by the substrate via tracer-derived acetly-CoA**	**13C-labeled fraction (m/z 281) (Σm)**	100.0	104.6	1	1	0.421230207	0.793482
	**13C Oleate labeled by the substrate via tracer-derived acetly-CoA, with only 2 carbons labeled to reflect novel synthesis via the tracer substrate**	**Chain Elongation (m/z281) (m2/Σm)**	100.0	67.2	1	-1	0.219304144	0.245354
**Glucose (C1-C4 - m/z 242) (Media-CAS: 50-99-7)**	**13C Glucose labeled by the substrate tracer**	**13C-labeled fraction (m/z 242) (Σm)**	100.0	100.4	1	1	0.260088175	0.079474
	**Percentage of 13C Glucose labeled by the substrate tracer**	**13C-m2 (m/z242) (m2/Σm)**	100.0	99.7	1	-1	0.24258539	0.059639
**RNA-ribose (C1-C4 - m/z 242) (Pellet-CAS:50-69-1)**	**13C-labeled ribose labeled by the substrate tracer and derived via the oxidative branch of the Pentose cycle**	**13C-labeled fraction (m/z 242) (Σm)**	100.0	6.6	1	-1	0.239180973	0.087109
**RNA-ribose (C3-C5 - m/z 217) (Pellet-CAS:50-69-1)**	**13C-labeled ribose labeled by the substrate tracer and derived via the non-oxidative branch of the Pentose cycle**	**13C-labeled fraction (m/z 217) (Σm)**	100.0	20.9	1	-1	0.237235359	0.102794

55.0	< 64 %
65.0	65 % - 78
80.0	79 % - 92
95.0	93 % - 106
110.0	107 % - 12
130.0	121 % - 13
140.0	135 % - 14
155.0	> 150 %
200.0	

55.0	65.0	80.0	95.0	110.0	130.0	140.0	155.0
< 64 %	65 % - 78 %	79 % - 92	93 % - 106 %	107 % - 120 %	121 % - 13	135 % - 14	> 150 %
