# Supplementary material for: Acidosis induces reprogramming of cellular metabolism to mitigate oxidative stress
Source: Cancer Metab. 2013 Dec 23;1:23. doi: 10.1186/2049-3002-1-23 (PMC4178214; doi:10.1186/2049-3002-1-23)
Supplement: Additional file 1: Table S1 — Listing of all primers and small interfering (si)RNAs used in this manuscript. Table S2-S7: Oncoisobolome and EZTop tables containing all relative and absolute measurements for all metabolites profiled in the glucose (Tables S2 and S3), glutamine (Tables S4 and S5) and palmitate (Tables S6 and S7) tracer studies. Metabolic profiles of MCF-7 cells in response to control (pH 7.4) or acidic (pH 6.7) conditions after 24 h of culture were obtained via SiDMAP analysis using [1,2-13C2]-d-glucose tracer, [U-13C2]-d-glutamine tracer, and [1,2-13C2]-palmitate tracer. Measured metabolites are as indicated, with identities determined and listed via Mn/Σm: isotopomer/13C labeled fraction as SUM(m1 + m2 + .. + mn). Σmn: molar enrichment (ME) 13C content as SUM(1 × m1 + 2 × m2 + .. + n × mn) (Lee et al.) (n = 4). Error bars are mean ± SD, P values as indicated (*P ≤0.05, **P ≤0.001, ***P ≤0.0001). [file 2049-3002-1-23-S1.zip › 1313128875104571_additional file 6_Table S6.pdf]

MCF-7	Palmitate Tracer	Absolute Measurements	pH	
Metabolite	Summary	Isotopomer Fragment Dimension	7.40	6.70
Complete Tracer Oxidation		13CO2 production	24.5 (±0.2422)	25.1 (±0.285)*
Extracellular Lactate (m/z 328)	Total 13C Lactate Pool	13C-labeled fraction (Σm)	1.17 (±0.0039)	0.09 (±0.0035)*
	13C lactate derived from incomplete TCA cycling	13C-m1 (m/z328) (m1/Σm)	8.84 (±0.387)	91.4 (±3.73)*
	13C Lactate derived from malate shuttling and incomplete TCA cycling	13C-m2 (m/z328) (m2/Σm)	82.9 (±0.43)	7.92 (±3.43)*
	Lactate-Derived from direct export of labeled oxaloacetate from the TCA cycle	13C-m3 (m/z328) (m3/Σm)	8.26 (±0.047)	0.703 (±0.304)*
Extracellular Glutamate (c2-c5) (m/z 198)	13C Glutamate labeled by the tracer substrate via the TCA cycle	13C-labeled fraction (m/z198) (Σm)	1.69 (±0..322)	1.80 (±0.642)*
	13C Glutamate labeled fraction produced from tracer-derived acetyl-CoA and lost via glutamine-ketoglutaric aminotransferase from the TCA cycle	13C-m2 (m/z198) (m2/Σm)	24.00 (±2.39)	23.72 (±2.53)*
	13C Glutamate-labeled fraction produced via tracer-labeled oxaloacetate and acetyl-CoA, then lost via glutamate transaminase II when the cycle is cut short.	13C-m4 (m/z198) (m4/Σm)	19.2 (±2.88)	24.46 (±0.848)*
Intracellular Palmitate (C:16)	13C Palmitate labeled by the substrate via labeled acetly-CoA	13C-labeled fraction (m/z 270) (Σm)	5.795 (±1.238)	3.941 (±0.4416)
	13C Palmitate labeled by the substrate via tracer-derived acetly-CoA, with only 2 carbons labeled to reflect novel synthesis via the tracer substrate	Chain Elongation (m/z270) (m2/Σm)	23.97 (±4.82)	25.12 (±4.596)
	Percentage of total13C Palmitate labeled by the substratewhich represents newly synthesized palmitate	Fraction of New Synthesis (FNS) (% of Total)	4.228 (±0.691)	4.029 (±0.517)
	13C Acetyl CoA labeled by the substrate	Ace-CoA (% of Total)	6.374 (±1.662)	5.347 (±2.072)
	13C Palmitate with one two carbon chain removed	One Chain Shortening	4.996 (±0.413)	5.196 (±0.873)
	Uniformally labeled 13C palmitate	U_13C_Palmitate	19.95 (±2.72)	13.58 (±1.771)
Intracellular Oleate (C18-1)	13C Oleate labeled by the substrate via tracer-derived acetly-CoA	13C-labeled fraction (m/z 281) (Σm)	47.62 (±6.807)	52.14 (±6.24)
	13C Oleate labeled by the substrate via tracer-derived acetly-CoA, with only 2 carbons labeled to reflect novel synthesis via the tracer substrate	Chain Elongation (m/z281) (m2/Σm)	1.875 (±0.237)	2.035 (±0.537)
Intracellular RNA-ribose (C1-C4) m/z 242	13C-labeled ribose labeled by the substrate tracer and derived via the oxidative branch of the Pentose cycle	13C-labeled fraction (m/z 242) (Σm)	0.0686 (±0.0087)	0.221 (±0.0413)
