# Supplementary material for: Acidosis induces reprogramming of cellular metabolism to mitigate oxidative stress
Source: Cancer Metab. 2013 Dec 23;1:23. doi: 10.1186/2049-3002-1-23 (PMC4178214; doi:10.1186/2049-3002-1-23)
Supplement: Additional file 1: Table S1 — Listing of all primers and small interfering (si)RNAs used in this manuscript. Table S2-S7: Oncoisobolome and EZTop tables containing all relative and absolute measurements for all metabolites profiled in the glucose (Tables S2 and S3), glutamine (Tables S4 and S5) and palmitate (Tables S6 and S7) tracer studies. Metabolic profiles of MCF-7 cells in response to control (pH 7.4) or acidic (pH 6.7) conditions after 24 h of culture were obtained via SiDMAP analysis using [1,2-13C2]-d-glucose tracer, [U-13C2]-d-glutamine tracer, and [1,2-13C2]-palmitate tracer. Measured metabolites are as indicated, with identities determined and listed via Mn/Σm: isotopomer/13C labeled fraction as SUM(m1 + m2 + .. + mn). Σmn: molar enrichment (ME) 13C content as SUM(1 × m1 + 2 × m2 + .. + n × mn) (Lee et al.) (n = 4). Error bars are mean ± SD, P values as indicated (*P ≤0.05, **P ≤0.001, ***P ≤0.0001). [file 2049-3002-1-23-S1.zip › 1313128875104571_additional file 7_Table S7.pdf]

MCF-7	Palmitate Tracer	Relative Measurements	pH		Statistical Tests			
Metabolite	Summary	Isotopomer Fragment Dimension	7.40	6.70	R square	Correlation	T test 1_tai	F test
Complete Tracer Oxidation		13CO2 production	100.0	102.4	1	1	0.5	1
Extracellular Lactate (m/z 328)	Total 13C Lactate Pool	13C-labeled fraction (Σm)	100.0	7.7	1	-1	0.245794	0.033772
	13C lactate derived from incomplete TCA cycling	13C-m1 (m/z328) (m1/Σm)	100.0	1033.9	1	1	0.250418	0.003339
	13C Lactate derived from malate shuttling and incomplete TCA cycling	13C-m2 (m/z328) (m2/Σm)	100.0	9.6	1	-1	0.245708	0.034467
	Lactate-Derived from direct export of labeled oxaloacetate from the TCA cycle	13C-m3 (m/z328) (m3/Σm)	100.0	8.5	1	-1	0.245756	0.034074
Extracellular Glutamate (c2-c5) (m/z 198)	13C Glutamate labeled by the tracer substrate via the TCA cycle	13C-labeled fraction (m/z198) (Σm)	100.0	106.5	1	1	0.322752	0.458198
	13C Glutamate labeled fraction produced from tracer-derived acetyl-CoA and lost via glutamine-ketoglutaric aminotransferase from the TCA cycle	13C-m2 (m/z198) (m2/Σm)	100.0	98.8	1	-1	0.177886	0.566058
	13C Glutamate-labeled fraction produced via tracer-labeled oxaloacetate and acetyl-CoA, then lost via glutamate transaminase II when the cycle is cut short.	13C-m4 (m/z198) (m4/Σm)	100.0	127.4	1	1	0.264551	0.113516
Intracellular Palmitate (C:16)	13C Palmitate labeled by the substrate via labeled acetly-CoA	13C-labeled fraction (m/z 270) (Σm)	100.0	68.0	1	-1	0.23792	0.097273
	13C Palmitate labeled by the substrate via tracer-derived acetly-CoA, with only 2 carbons labeled to reflect novel synthesis via the tracer substrate	Chain Elongation (m/z270) (m2/Σm)	100.0	104.8	1	1	0.358849	0.600937
	Percentage of total13C Palmitate labeled by the substratewhich represents newly synthesized palmitate	Fraction of New Synthesis (FNS) (% of Total)	100.0	95.3	1	-1	0.172513	0.610859
	13C Acetyl CoA labeled by the substrate	Ace-CoA (% of Total)	100.0	83.9	1	-1	0.226076	0.192059
	13C Palmitate with one two carbon chain removed	Chain Shortening (m/z270) (m2/Sm)	100.0	104.0	1	1	0.3888	0.699032
	Uniformly labeled 13C palmitate	U_13C_Palmitate (m/z 270)	100.0	68.1	1	-1	0.237897	0.097465
Intracellular Oleate (C18-1)	13C Oleate labeled by the substrate via tracer-derived acetly-CoA	13C-labeled fraction (m/z 281) (Σm)	100.0	109.5	1	1	0.295803	0.321497
	13C Oleate labeled by the substrate via tracer-derived acetly-CoA, with only 2 carbons labeled to reflect novel synthesis via the tracer substrate	Chain Elongation (m/z281) (m2/Σm)	100.0	108.5	1	1	0.301968	0.355843
Intracellular RNA-ribose (C1-C4) m/z 242	13C-labeled ribose labeled by the substrate tracer and derived via the oxidative branch of the Pentose cycle	13C-labeled fraction (m/z 242) (Σm)	100.0	322.2	1	1	0.251758	0.014035

55.0	< 64 %
65.0	65 % - 78 %
80.0	79 % - 92 %
95.0	93 % - 106 %
110.0	107 % - 120 %
130.0	121 % - 134 %
140.0	135 % - 149 %
155.0	> 150 %
200.0	

55.0	65.0	80.0	95.0	110.0	130.0	140.0	155.0
< 64 %	65 % - 78 %	79 % - 92	93 % - 106	107 % - 12	121 % - 13	135 % - 14	> 150 %
