# Supplementary figures and images for: Acidosis induces reprogramming of cellular metabolism to mitigate oxidative stress
Source: Cancer Metab. 2013 Dec 23;1:23. doi: 10.1186/2049-3002-1-23 (PMC4178214; doi:10.1186/2049-3002-1-23)

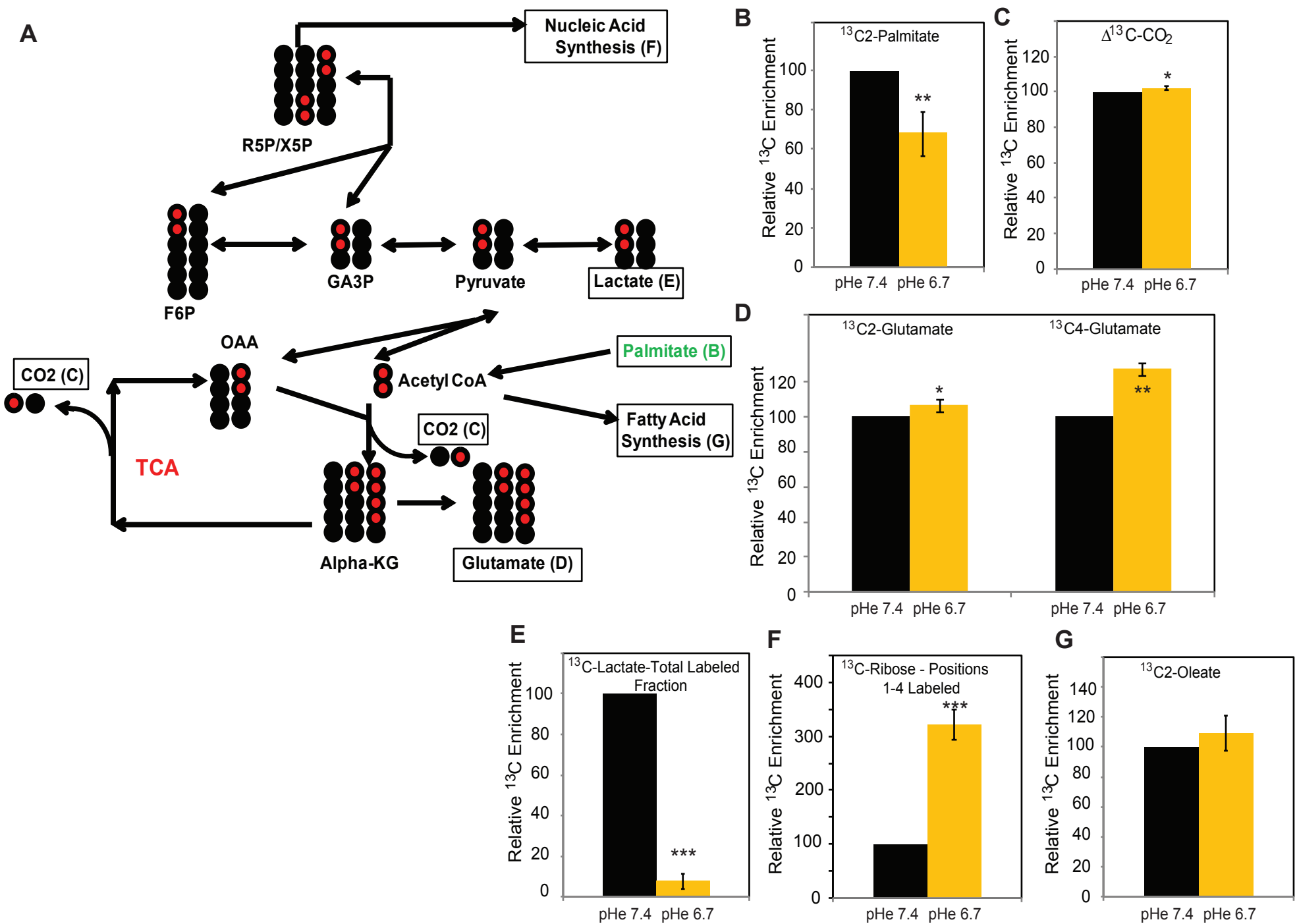

Figure S1

Supplement: Additional file 2: Figure S1 — The use of 13C palmitate isotope tracer to analyze glutamine metabolism under acidosis. (A) Schematic graph indicating the measured metabolites (and corresponding panels) resulting from the uniformly 13C labeled palmitate tracer under control or acidosis conditions. The relevant substrate tracer is indicated in green, 13C labeled carbons are indicated in red (normal carbon atoms are black). (B-G). Relative 13C enrichment in the palmitate (B), CO2 (C), glutamate (D), lactate (E), ribonucleic acids (F) and oleate (G) under control or acidosis conditions. Glutamate (D) is presented as both the 2 (C2 (E)) and 4 (C4 (B)) labeled carbon subpools. Lactate (E) is presented as the total 13C-labeled lactate pool. Ribonucleic acids (F) are presented as the 13C positions 1 to 4 subpool. Fatty acids (B,G) are presented as 2-carbon 13C-labeled palmitate (B) and oleate (G). Error bars are mean ± SD, significant P values are indicated (*P ≤0.05, **P ≤0.01, ***P ≤0.001). [file 2049-3002-1-23-S2.pdf]

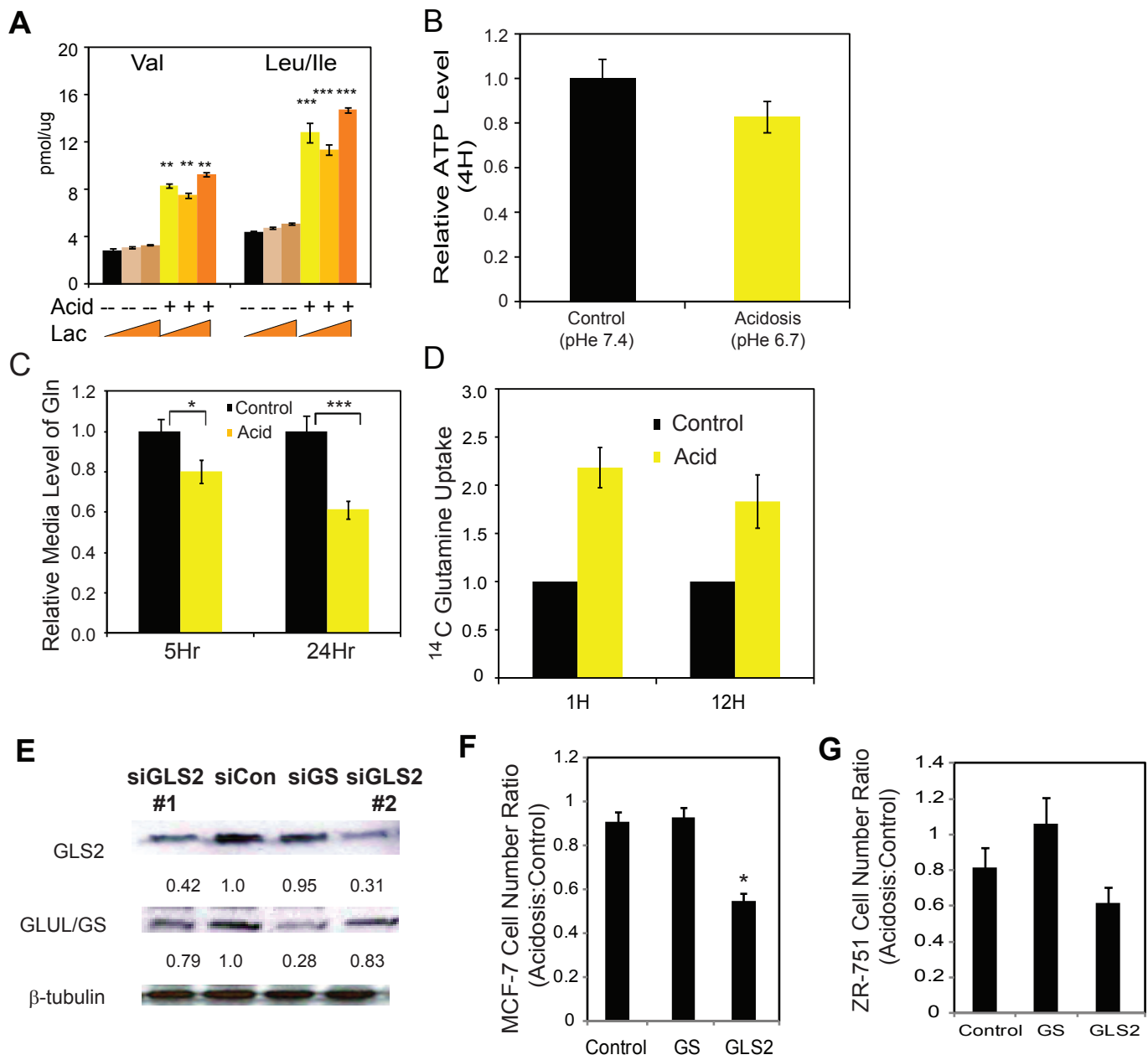

Figure S2

Supplement: Additional file 3: Figure S2 — Essential role of glutaminolysis under acidosis. (A) The intracellular levels of Val and Leu/Ile under indicated conditions of acidosis or lactic acidosis conditions (n = 3). (B) Normalized cellular ATP levels in MCF-7 cells under control or acidosis conditions after 4 h. (C) Measurements of glutamine in cell culture media at 5 and 24 h after exposure to acidosis. (D)14C-glutamine levels in cell pellets under control or acidosis conditions in MCF-7 cells at 1 h and 12 h. (E) Levels of the indicated proteins in the glutamine/glutamate metabolism pathways after the gene silencing by respective small interfering (si)RNAs. (F,G) Relative cell numbers (as a ratio of acidosis/control) of MCF-7 (F) and ZR-75-1 (G), determined by propidium iodide staining, when the indicated genes were silenced under normal or acidosis conditions (n = 3). Error bars are mean ± SD, significant P values are indicated (*P ≤0.05, **P ≤0.01, ***P ≤0.001). [file 2049-3002-1-23-S3.pdf]

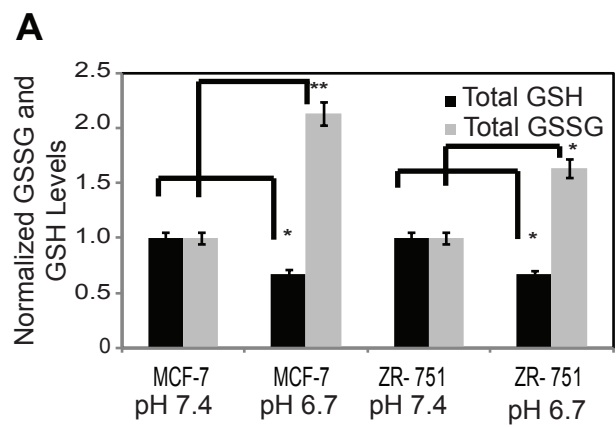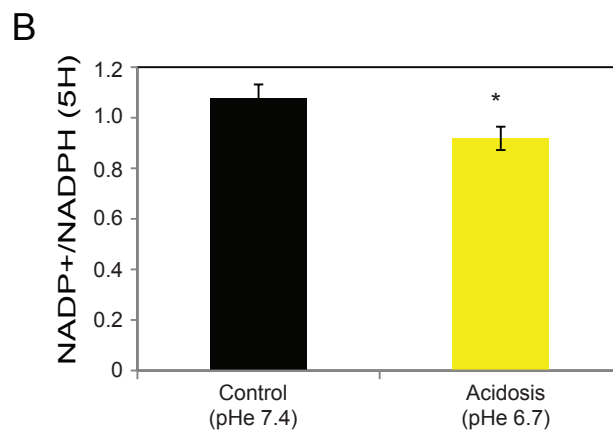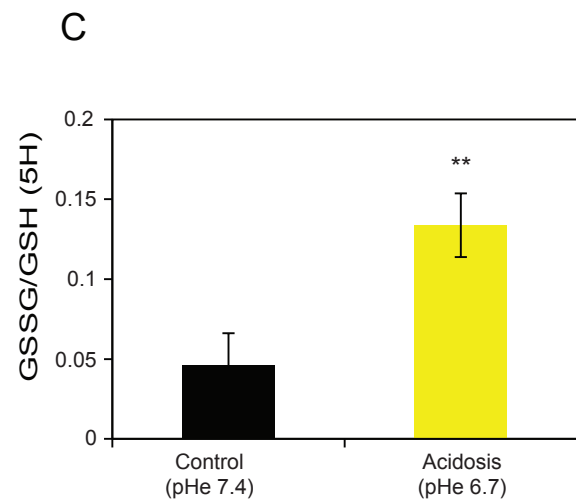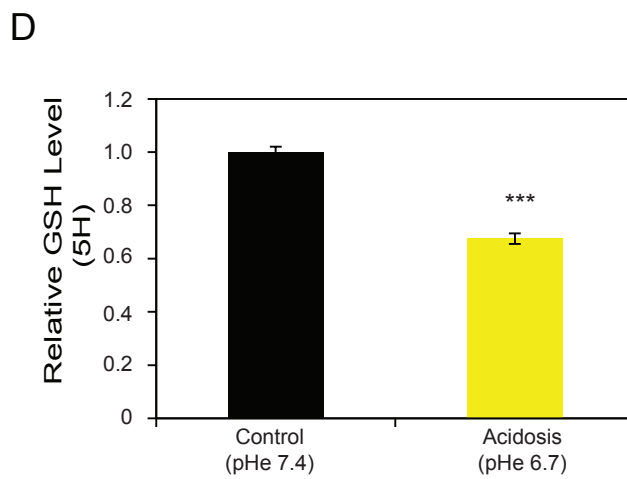

Figure S3

Supplement: Additional file 4: Figure S3 — Effects of acidosis on glutathione (GSH)/glutathione disulfide (GSSG) and NADP+/nicotinamide adenine dinucleotide phosphate (NADPH) after 5 h of exposure (A) Normalized total GSH and GSSG levels for MCF-7 and ZR-75-1 cells under control or acidosis conditions (pH 6.7). (B-D) NADP/NADPH ratio, GSSG/GSH ratio, normalized total GSH levels of MCF-7 cells after 5 h of either control or acidosis conditions. Error bars are mean ± SD, significant P values are indicated (*P ≤0.05, **P ≤0.01, ***P ≤0.001). [file 2049-3002-1-23-S4.pdf]

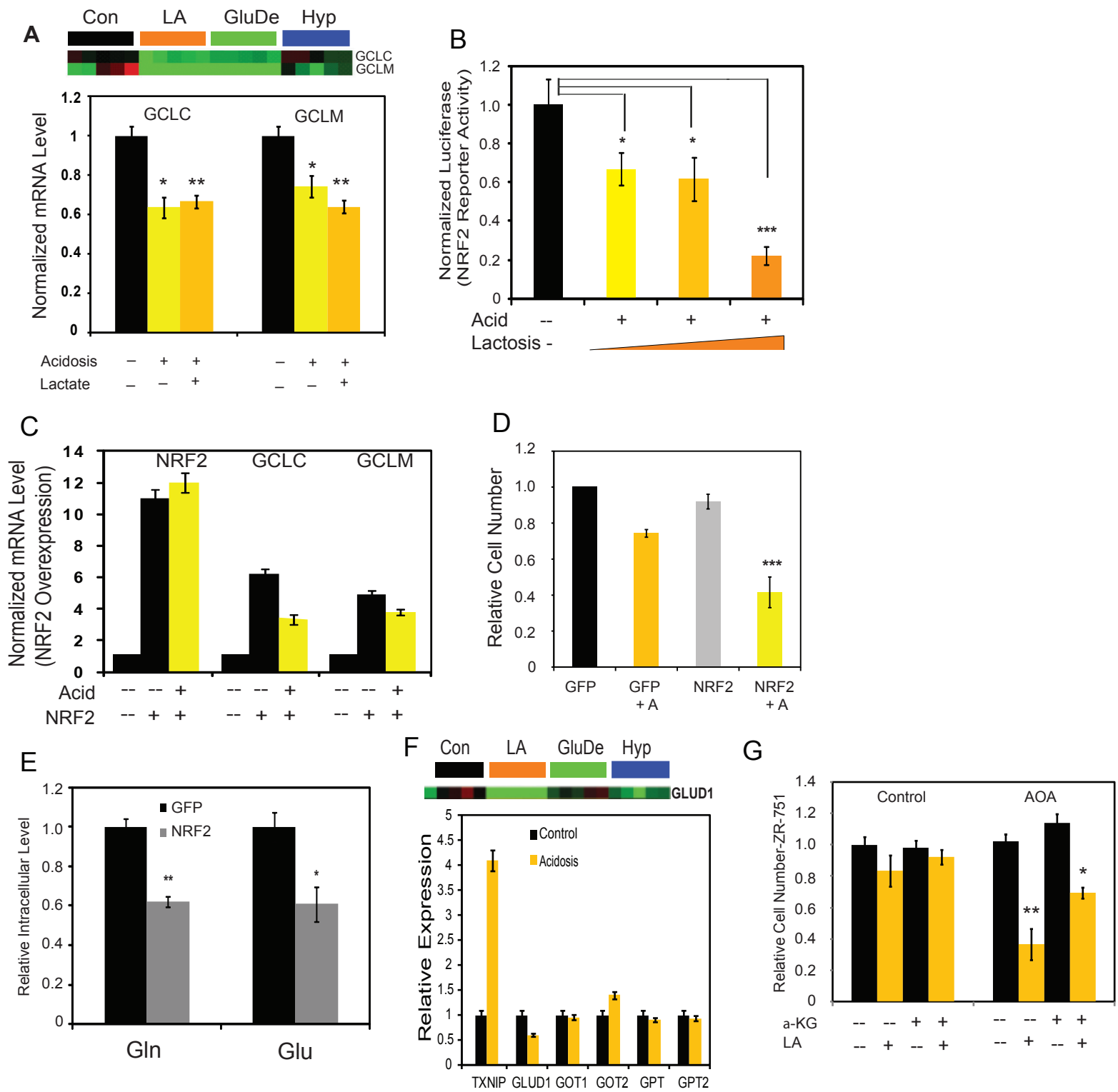

Figure S4

Supplement: Additional file 5: Figure S4 — Acidosis reduced nuclear factor erythroid 2-related factor 2 (NRF2) activities and increased levels of ROS. (A) Relative mRNA abundance, determined by microarray and quantitative real-time PCR (qPCR), for the indicated genes under control or lactic acidosis conditions. (B) Relative NRF2 activity, as determined by luciferase reporter, for MCF-7 cells exposed to control or lactic acidosis conditions. (C) Relative mRNA levels of the indicated genes, after green fluorescent protein (GFP) or NRF2 overexpression, as determined by qPCR. (D) Relative cell numbers 48 h after the expression of GFP or NRF2 in MCF-7 cells under control or acidosis conditions. (E) Intracellular normalized levels of glutamine and glutamate in MCF-7 cells that have been transfected with GFP or NRF2 expression constructs. (F) Relative transcript abundance, determined by microarray and qPCR, for the indicated genes under control, acidosis (qPCR only) or lactic acidosis conditions. (G) Relative cell numbers for ZR-75-1 cells treated with 0.2 mM amino-oxyacetate (AOA) or under control or acidosis conditions. Indicated cells are also supplemented with 700 uM dimethyl α-ketoglutarate (α-KG) (n = 4). Error bars are mean ± SD, significant P values are indicated (*P ≤0.05, **P ≤0.01, ***P ≤0.001). [file 2049-3002-1-23-S5.pdf]

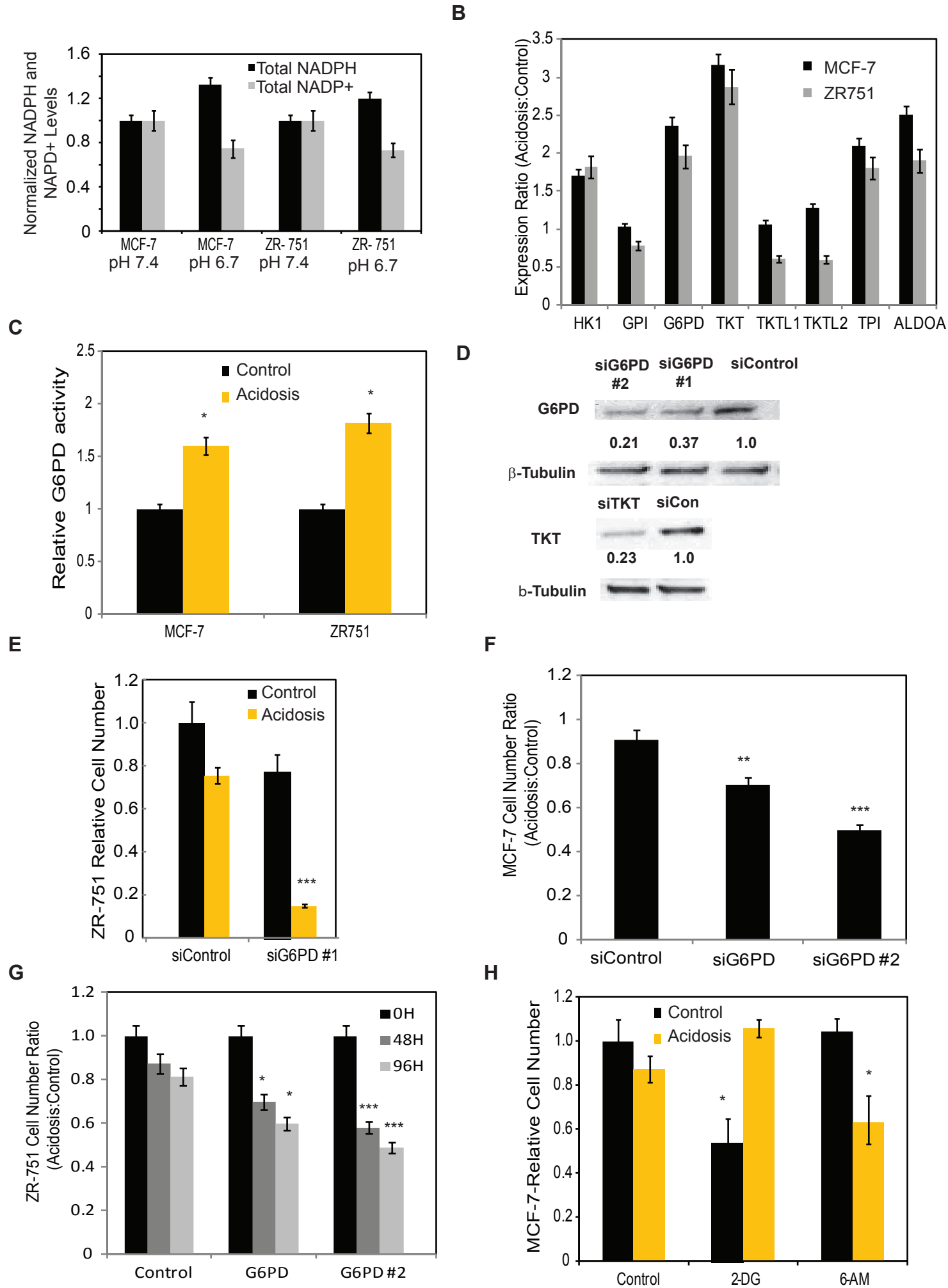

Figure S5

Supplement: Additional file 6: Figure S5 — The effects of acidosis on the expression of genes that encode proteins in the pentose phosphate pathways (PPPs). (A) Normalized NADP + and nicotinamide adenine dinucleotide phosphate (NADPH) levels in MCF-7 and ZR-75-1 cells under control and acidosis conditions. (B) The acidosis-induced change of mRNA expression for the indicated genes in MCF-7 and ZR-75-1 cells. (C) Relative glucose-6-phosphate dehydrogenase (G6PD) activity in MCF-7 and ZR-75-1 cells under control or acidosis conditions. (D) Protein levels of G6PD and transketolase 1 (TKT1) in MCF-7 cells transfected by control (siControl), two small interfering (si)RNAs targeting G6PD (siG6PD), or siRNA targeting TKT1 (siTKT1). (E) Relative cell numbers of ZR-75-1 cells transfected with control or siRNA targeting G6PD under control or acidosis conditions. (F,G) The change in cell numbers of MCF-7 (F) and ZR-75-1 (G) cells, under acidosis, treated with the indicated siRNAs determined by propidium iodide staining. (H) Relative cell numbers of MCF-7 cells that have been treated with dimethylsulfoxide (DMSO), 2-deoxyglucose (2-DG) or 6-aminonicotinamide (6-AM) under control or acidosis conditions (n = 4). Error bars are mean ± SD, significant P values are indicated (*P ≤0.05, **P ≤0.01, ***P ≤0.001). [file 2049-3002-1-23-S6.pdf]

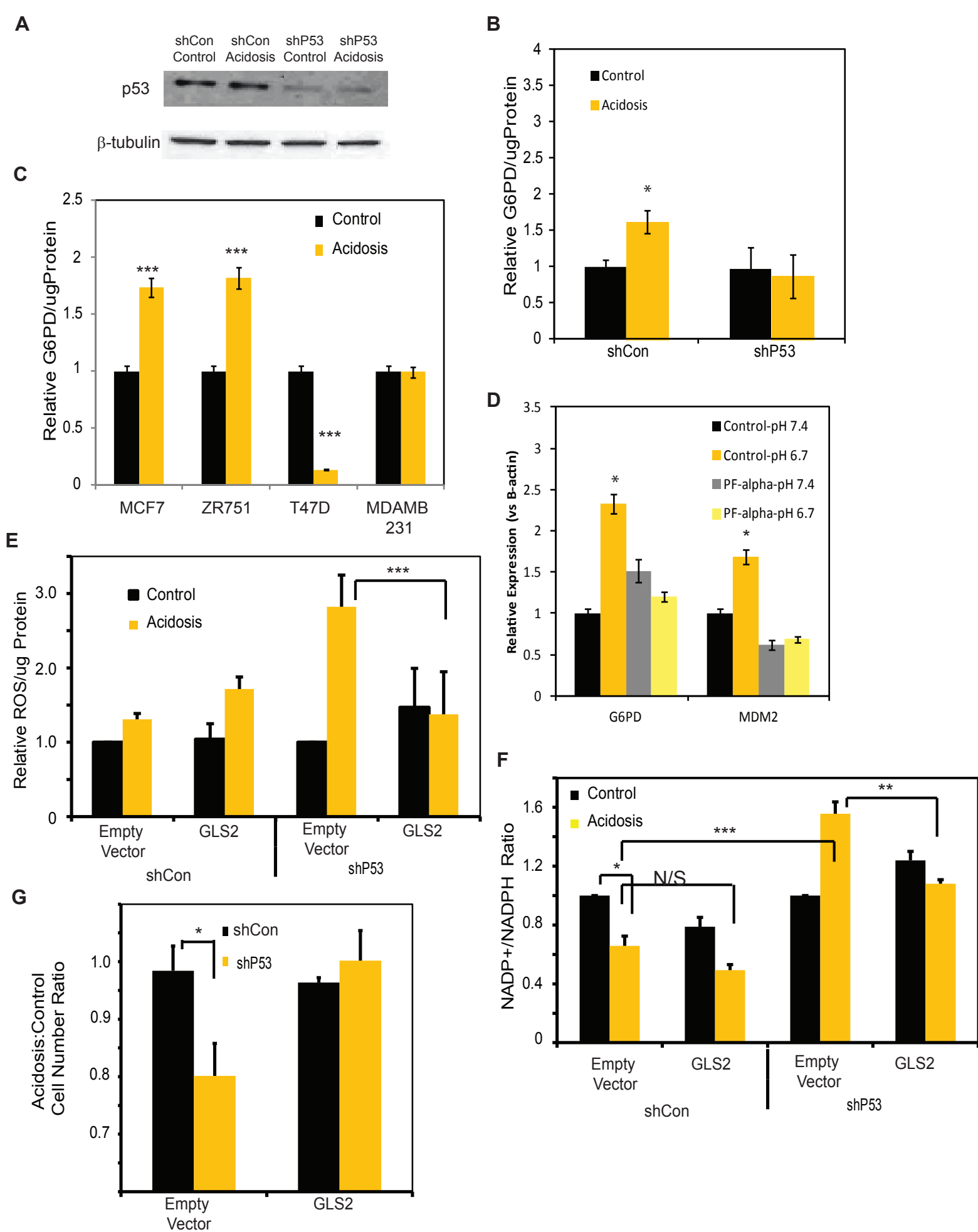

Figure S6

Supplement: Additional file 7: Figure S6 — The role of p53 in the acidosis response. (A) Protein levels of p53 in MCF-7 cells expressing shp53 or shControl. (B) Glucose-6-phosphate dehydrogenase (G6PD) activity in MCF-7 cells expressing shCon or shP53 exposed to either control or acidosis conditions. (C) G6PD activity in the indicated cell lines exposed to either control or acidosis conditions. (D) mRNA expression of the indicated genes exposed to either control or pifithrin-α under normal or acidosis conditions. (E-G) normalized ROS levels (E), NADP+/nicotinamide adenine dinucleotide phosphate (NADPH) ratio (F) and normalized cell numbers (G) in the shControl or shP53 MCF-7 cells when they were transfected with either GLS2 or empty expression vector, under control or acidosis conditions. Error bars are mean ± SEM, P values as indicated (*P ≤0.05, **P ≤0.001, ***P ≤0.0001). [file 2049-3002-1-23-S7.pdf]
